# Epigenetic regulation of *HOXA2* expression affects tumor progression and predicts breast cancer patient survival

**DOI:** 10.1038/s41418-024-01430-2

**Published:** 2025-01-20

**Authors:** Fatima Domenica Elisa De Palma, Jonathan G. Pol, Vincent Carbonnier, Sarah Adriana Scuderi, Deborah Mannino, Léa Montégut, Allan Sauvat, Maria Perez-Lanzon, Elisabet Uribe-Carretero, Mario Guarracino, Ilaria Granata, Raffaele Calogero, Valentina Del Monaco, Donatella Montanaro, Gautier Stoll, Gerardo Botti, Massimiliano D’Aiuto, Alfonso Baldi, Valeria D’Argenio, Roderic Guigó, René Rezsohazy, Guido Kroemer, Maria Chiara Maiuri, Francesco Salvatore

**Affiliations:** 1 CEINGE-Biotecnologie Avanzate Franco Salvatore, Naples, Italy; 2 Department of Molecular Medicine and Medical Biotechnologies, University of Naples Federico II, Naples, Italy; 3 Team «Metabolism, Cancer & Immunity », Centre de Recherche des Cordeliers, INSERM UMRS1138, Sorbonne Université, Université de Paris, Paris, France; 4 Metabolomics and Cell Biology Platforms, Gustave Roussy Cancer Campus, Villejuif, France; 5 Department of Chemical, Biological, Pharmaceutical and Environmental Sciences, University of Messina, Messina, Italy; 6 Centro de Investigacion Biomedica en Red de Enfermedades Neurodegenerativas (CIBERNED), Depto. Bioquimica y Biologia Molecular y Genetica, Facultad de Enfermeria y Terapia Ocupacional, Caceres, Spain; 7 University of Cassino and Southern Lazio, Cassino, Italy; 8 National Research University Higher School of Economics, Moscow, Russia; 9 National Research Council, Inst. for High-Performance Computing and Networking, Naples, Italy; 10 Department of Molecular Biotechnology and Health Sciences, University of Torino, Torino, Italy; 11 Department of Senology, Istituto Nazionale Tumori—IRCCS Fondazione Pascale, Naples, Italy; 12 Department of Environmental, Biological and Pharmaceutical Sciences and Technologies, University of Campania “Luigi Vanvitelli”, Caserta, Italy; 13 Department of Human Sciences and Quality of Life Promotion, San Raffaele Open University, Rome, Italy; 14 Universitat Pompeu Fabra (UPF), Barcelona, Spain; 15 Bioinformatics and Genomics, Centre for Genomic Regulation (CRG), Barcelona, Catalonia Spain; 16 Louvain Institute of Biomolecular Science and Technology, UCLouvain, Louvain-la-Neuve, Belgium; 17 Institut du Cancer Paris CARPEM, Department of Biology, Hôpital Européen Georges Pompidou, Assistance Publique-Hôpitaux de Paris, Paris, France; 18 Inter-University Center for multifactorial and multi genetic chronic human diseases, “Federico II”- Naples, Tor Vergata- Roma II and Chieti-Pescara Universities, Chieti-Pescara, Italy

**Keywords:** Diagnostic markers, Tumour biomarkers

## Abstract

Accumulating evidence suggests that genetic and epigenetic biomarkers hold potential for enhancing the early detection and monitoring of breast cancer (BC). Epigenetic alterations of the *Homeobox A2* (*HOXA2*) gene have recently garnered significant attention in the clinical management of various malignancies. However, the precise role of *HOXA2* in breast tumorigenesis has remained elusive. To address this point, we conducted high-throughput RNA sequencing and DNA methylation array studies on laser-microdissected human BC samples, paired with normal tissue samples. Additionally, we performed comprehensive in silico analyses using large public datasets: TCGA and METABRIC. The diagnostic performance of *HOXA2* was calculated by means of receiver operator characteristic curves. Its prognostic significance was assessed through immunohistochemical studies and Kaplan-Meier Plotter database interrogation. Moreover, we explored the function of *HOXA2* and its role in breast carcinogenesis through in silico, in vitro, and in vivo investigations. Our work revealed significant hypermethylation and downregulation of *HOXA2* in human BC tissues. Low *HOXA2* expression correlated with increased BC aggressiveness and unfavorable patient survival outcomes. Suppression of *HOXA2* expression significantly heightened cell proliferation, migration, and invasion in BC cells, and promoted tumor growth in mice. Conversely, transgenic *HOXA2* overexpression suppressed these cellular processes and promoted apoptosis of cancer cells. Interestingly, a strategy of pharmacological demethylation successfully restored *HOXA2* expression in malignant cells, reducing their neoplastic characteristics. Bioinformatics analyses, corroborated by in vitro experimentations, unveiled a novel implication of HOXA2 in the lipid metabolism of BC. Specifically, depletion of *HOXA2* leaded to a concomitantly decreased expression of *PPARγ* and its target *CIDEC*, a master regulator of lipid droplet (LD) accumulation, thereby resulting in reduced LD abundance in BC cells. In summary, our study identifies *HOXA2* as a novel prognosis-relevant tumor suppressor in the mammary gland.

## Introduction

Along with lung and colon cancer, breast cancer (BC) is one of the three most common cancers worldwide [1, 2]. BC is a complex and dynamic malignancy characterized by a high inter- and intra-tumoral heterogeneity that complicates its multidisciplinary clinical management [3]. BC is categorized into four subtypes based on the expression of the estrogen receptor (ER), progesterone receptor (PR), human epidermal growth factor receptor 2 (HER2), and the proliferation marker Ki67: luminal A, luminal B, HER2-positive (HER2^+^), and triple-negative breast cancer (TNBC) [1]. Each subgroup of BC is defined not only by molecular but also histological characteristics and is associated with a specific prognosis and treatment [1, 4, 5].

The management of cancer patients has benefited from the advent of diagnostic, prognostic, and predictive indicators, mainly of protein and nucleic acid nature [6–12]. In BC, the list of biomarker candidates expands at a sustained pace and includes some gene mutations and variations of the level of protein-coding or non-coding RNAs that display an interest in the diagnosis of the disease, in differentiating between BC subtypes, and/or in predicting the prognosis and therapy-responsiveness of patients [13–16].

*Homeobox* (*HOX)* genes have been explored as potential biomarkers in several types of tumors, including BC [17–19]. *HOX* genes play essential roles in embryo development and tissue homeostasis [19, 20]. Alterations in their expression, which is often epigenetically controlled (mainly by methylation at CpG sites), may contribute to the development and/or progression of mammary carcinomas through effects on cell-autonomous phenomena like autophagy and apoptosis as well as non-cell-autonomous processes such as angiogenesis, invasion and metastasis [19, 21, 22]. Notably, reduced expression of the transcription factor HOXA5 promotes breast tumorigenesis *via* several pathways. Thus, *HOXA5* downregulation (secondary to the hypermethylation of its promoter) leads to low expression of p53. In addition, *HOXA5* can induce apoptosis via a p53-independent mechanism, via the activation of caspases 2 and 8 [23, 24]. Moreover, *HOXA1* promotes the survival of malignant cells by activating the nuclear factor kappa-light-chain-enhancer of activated B cells (NF-κB) pathway *via* its binding to RBCK1/HOIL-1 and TRAF2 [25]. Elevated expression of *HOXA1* has been associated with poor prognosis and tumor progression in BC [26].

Reduced *HOXA2* expression and hypermethylation of its promoter can serve as a diagnostic and prognostic indicator for certain malignancies [27–32], as exemplified by prostate cancer [28], lung squamous cell carcinoma (SCC) [33] and colorectal cancer (CRC) [29]. However, the direct involvement of *HOXA2* in breast tumorigenesis has not yet been explored. In this framework, we explored: i) *HOXA2* gene expression and methylation status, ii) clinicopathological correlates of HOXA2 expression assessed by immunohistochemistry, iii) HOXA2 prognostic value, iv) the effects of *HOXA2* deregulation on malignant traits in vitro and in vivo, and v) the molecular mechanism through which *HOXA2* suppresses breast carcinogenesis.

## Results

### Transcriptome and gene methylome of patient breast tissues

**Fig. 1:**
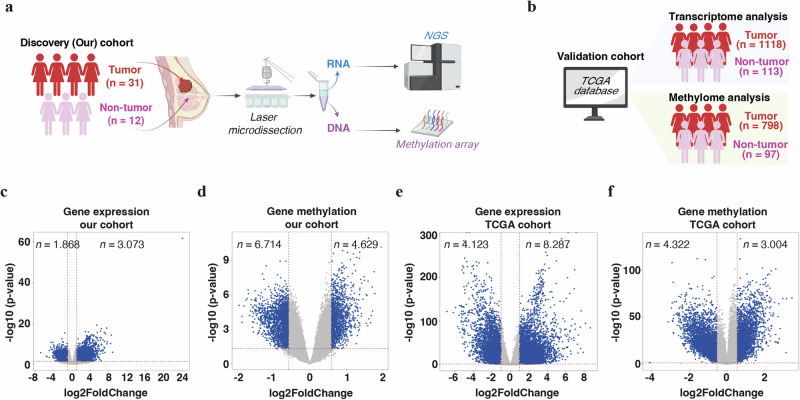
Transcriptome and DNA methylome profiling of human breast cancer tissues. Workflow of the experimental design to identify tumor suppressor genes in breast cancer (BC) via RNA-sequencing and DNA methylation assays using non-tumor/normal and malignant breast tissue specimens extracting data from our (discovery) cohort of breast patients (**a**), or from TCGA dataset (**b**). **a**, **b** were created with BioRender.com. Volcano plots of differentially expressed genes (**c**, **e**), and differentially methylated genes (**d**, **f**) (reported according to their log_2_ fold change and p-value in non-tumor/normal versus tumor breast samples), from our cohort of BC individuals (**c**, **d**) and from TCGA dataset (**e**, **f**). Of note, *p*-value ≤ 0.05 and |log_2_FC|≥ 1 for significant differentially expressed genes (**c**, **e**), and *p*-value ≤ 0.05 and |log_2_FC|≥ 0.5 for significant differentially methylated genes (**d**, **f**), were used as cut-off. Significant and insignificant modulations are in blue and grey, respectively. See Supplementary Tables S1–S5 for details. FC fold change; TCGA The Cancer Genome Atlas.

To identify novel genes and/or transcript sequences involved in breast tumorigenesis, we investigated the transcriptome and DNA methylome of breast tissue samples of surgery-operated patients (Fig. 1a, b).

First, we examined the gene expression pattern of 43 laser-microdissected breast tissue samples (31 tumor, 12 normal) using high-throughput RNA sequencing. Out of the 4 941 coding sequences differentially expressed (DE), 3 073 (62.2%) were highly (*p*-value ≤ 0.05, |log_2_FC| ≥ 1) up-regulated and 1 868 (37.8%) highly down-regulated in tumor versus normal breast tissues (Fig. 1c and Supplementary Table S1).

Promoter methylation has been involved in the emergence of cancer through the inactivation of tumor suppressor genes [34, 35]. Therefore, we investigated the genome-wide DNA methylation profile of the same cohort. Out of the 162 832 individual CpG islands differentially methylated (DM) (*p*-value ≤ 0.05, |log_2_FC| ≥ 0.5), 49.3% were hypermethylated and 50.7% hypomethylated in malignant versus normal breast tissues (Supplementary Fig. 1a and Supplementary Table S2). To identify genes with a strong DM status, we computed the mean CpG methylation value of each gene. Out of the 11 343 DM genes (*p*-value ≤ 0.05, |log_2_FC| ≥ 0.5), the majority (*n* = 6714; 59.2%) harbored reduced levels of methylation in malignant versus normal breast tissues (Fig. 1d, and Supplementary Table S3).

Next, to validate the results obtained in our investigational cohort, we interrogated the TCGA database (Fig. 1e, f, Supplementary Fig. S1b, and Supplementary Table S4, S5). Among the genes differentially expressed between normal and malignant TCGA samples (*n* = 12 410), 2 005 were also found dysregulated (*p*-value ≤ 0.05, |log_2_FC| ≥ 1) in our cohort (Supplementary Table S6). Similarly, 1103 genes demonstrated comparable modulation (*p*-value ≤ 0.05, |log_2_FC| ≥ 0.5) of their methylation status in our cohort and in TCGA (Supplementary Table S7).

These DE and DM gene signatures allowed segregation samples from healthy and cancer patients (Supplementary Fig. 1c–f and Supplementary Tables S1, S3–S5).

### *HOXA2* is hypermethylated and downregulated in human breast cancer

**Fig. 2:**
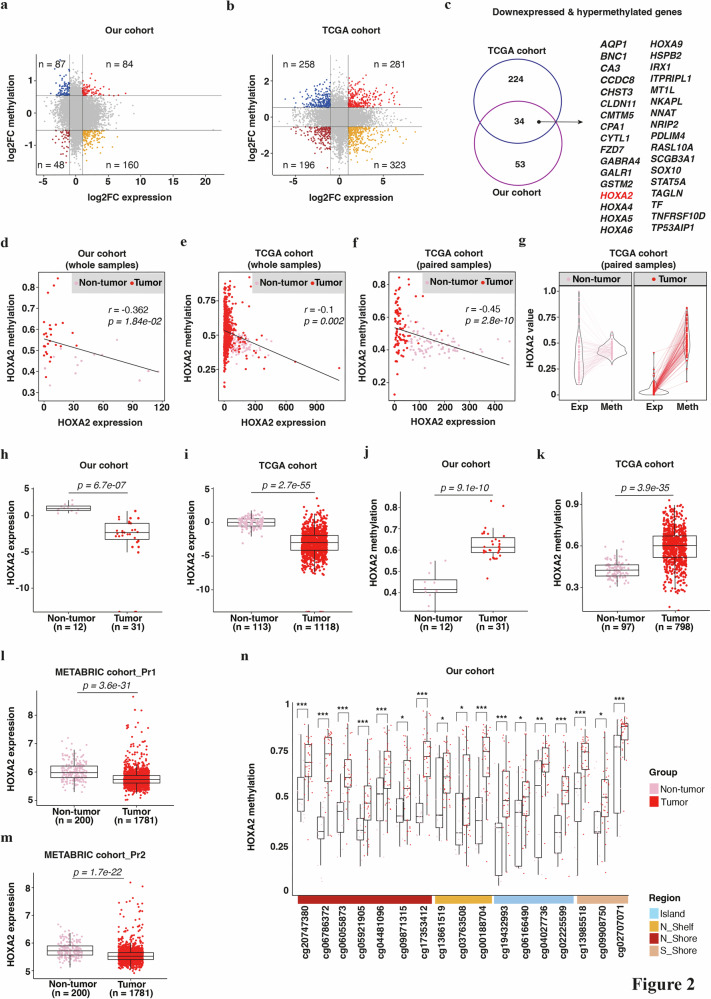
Reduced expression and promoter hypermethylation of *HOXA2* in breast cancer. Scatter plots displaying significant (*p*-value ≤ 0.05) log_2_ fold change (FC) values of differentially expressed (x-axis, |log_2_FC| ≥ 1) against differentially methylated (y-axis, |log_2_FC| ≥ 0.5) genes in non-tumor/normal versus tumor BC tissues from our cohort of samples (**a**) and from TCGA dataset (**b**). **c** Venn diagram showing overlapping hypermethylated and down-expressed genes in our cohort and TCGA datasets. The genes shared between the two cohorts of samples are listed. Plots showing the correlation between the methylation and expression of *HOXA2* in non-tumor/normal and tumor breast samples in the whole cohort (**d**, **e**) or more specifically in patients where matched tumor and adjacent non-tumor specimens were available (**f**), from our cohort (**d**) or TCGA dataset (**e**, **f**). **g** Violin plot exhibiting the comparison of the expression (Exp) and methylation (Meth) of HOXA2 using individual breast cancer tissue samples and their adjacent non-tumor counterparts (paired samples) from TCGA dataset. **d**–**g** Individual values are reported as transcript per million (TPM) for expression, and as beta values for methylation. Coefficients of correlations (R) and significance are displayed on the graphs. **h**–**n** Boxplots illustrating the individual level of expression (**h**, **i**, **l**, **m**) and methylation (**j**, **k**) of *HOXA2* gene in non-tumor/normal and malignant breast tissues of our cohort of patients (**h**, **j**), and derived from TCGA (**i**, **k**) and METABRIC (**l**, **m**) datasets. Box and whisker plots illustrate the level of expression of *HOXA2* as log2 (FPKM + 10^-4^) and of *HOXA2* methylation as the average of mean beta values (0 = unmethylated, 1 = methylated) for each breast tissue sample. Corresponding data of the statistical analyses are shown in Supplementary Tables S1 and S4–S5. The differential expression of *HOXA2* was measured using two probe sets (Pr1 in (**l**); Pr2 in (**m**)) in METABRIC dataset. Bars represent mean values ± standard deviation. Data show median, quartiles, and individual values. **n** Boxplot showing differential levels of methylation within different significant CpG loci of *HOXA2* in non-tumor/normal and breast tumor samples derived from our cohort of patients. Each CpG site (according to the Infinium 450k BeadChip probe classification = CpG island regions, CpG-island shores (N and S), and CpG-island shelves) is color-coded in the right bar above. Individual values are expressed as beta values. See Supplementary Table S11 for complementary data. *p-value ≤ 0.05, **p-value ≤ 0.01, ***p-value ≤ 0.001. CpG Cytosine phosphate Guanine; FPKM fragments per kilobase of exon per million fragments mapped; METABRIC Molecular Taxonomy of Breast Cancer International Consortium; Pr1 probe set 1; Pr2 Probe set 2; TCGA The Cancer Genome Atlas.

Since DNA methylation is one of the most frequently observed causes of tumor suppressor inactivation, we performed an integrative analysis of DNA methylation and RNA-seq gene expression to identify novel tumor suppressor genes. Notably, we screened for genes showing both high levels of methylation (log_2_FC ≥ 0.5) and low levels of expression (log_2_FC ≤ 1) in BC samples in our cohort and in TCGA database (Fig. 2a, b, and Supplementary Tables S8, S9). Among the significant (*p*-value ≤ 0.05) genes identified, 34 genes were hypermethylated and less expressed in both cohorts (Fig. 2c, Supplementary Table S10). The majority of these candidate tumor suppressor genes (e.g., *BCN1*, *CCND1*, *HOXA4*, *HOXA5*, *HOXA9*, and *STAT5A*) were already described in the literature in BC [36–40].

We focused on the gene encoding the transcription factor homeobox A2 (*HOXA2*, Ensembl ID: ENSG00000105996), whose role in BC is not well investigated. *HOXA2* demonstrated a significant correlation between hypermethylation and reduced expression in our cohort of BC (Fig. 2d), as well as in the TCGA dataset (Fig. 2e). This correlation was particularly obvious when restricting the analysis to paired tumor and non-tumor samples from the TCGA database (Fig. 2f). At the individual level, the expression of *HOXA2* in non-tumor tissues coincided with a median level of methylation, whereas the absence of *HOXA2* mRNA was recurrent in tumor tissues and mostly associated with DNA hypermethylation (Fig. 2g). More precisely, *HOXA2* showed a 2- and 4-fold decrease of its expression (Fig. 2h, i) and a concomitant 40% and 50% increase of its methylation (Fig. 2j, k) in BC when compared to normal breast tissues in our cohort and TCGA, respectively (Supplementary Tables S1, S3–S7). In addition, the altered abundance of *HOXA2* in BC tissues was not only confirmed at the mRNA level by RT-qPCR on the same cohort of samples (Supplementary Fig. S1g) but also using the METABRIC dataset (Fig. 2 l,m).

We also estimated differences in the *HOXA2* gene methylation level in normal and malignant breast tissues across the CpG islands and their proximal shelves and distal shores. Out of the 34 CpG regions screened, *HOXA2* was significantly hypermethylated (*p*-value ≤ 0.05, |log_2_FC| ≥ 0.5) in 17 loci (Fig. 2n, Supplementary Table S11). This pattern was confirmed in 11 of the 17 loci in TCGA dataset (Supplementary Fig. S1h, Supplementary Table S12).

In conclusion, it appears that *HOXA2* promoter hypermethylation correlates with a reduction of *HOXA2* mRNA in breast tumor samples.

### Clinical role of *HOXA2* deregulation in breast cancer

**Fig. 3:**
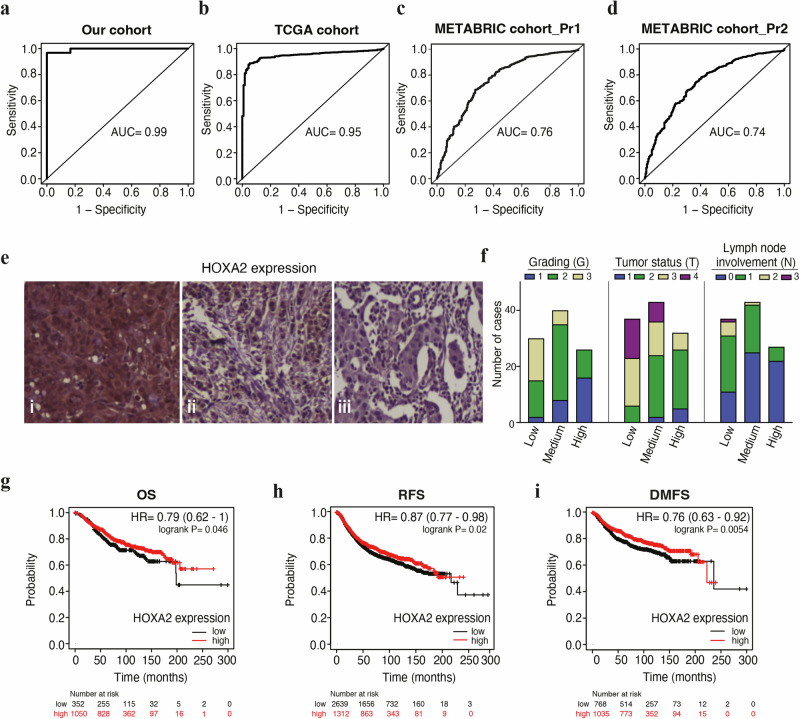
Expression level of HOXA2 is a diagnostic and prognostic indicator in breast cancer. Diagnostic value of HOXA2 expression level in our cohort of breast samples (**a**), TCGA dataset (**b**), and METABRIC dataset (**c**, **d**) using two probe sets for HOXA2 transcript (**c**, probe set 1; **d**, probe set 2) through ROC curve analysis. **e** Representative immunohistochemistry images showing the staining of HOXA2 protein in human breast tissues (40x). From left to right panel: (i) high expression (more than 20% of positive cells), (ii) medium expression (from 1% to 20% of positive cells) and (iii) low expression (less than 1% of positive cells) of HOXA2 protein. **f** Graphical representation of the different expression levels of HOXA2 in 96 BC tissues obtained according to histological (G, grading) and clinical (T, tumor status and N, lymph node involvement) parameters. See Supplementary Table S13 for complementary data. Kaplan-Meier analysis of **g** OS, **h** RFS, and **i** DMFS in BC patients expressing high (red curves) versus low (black curves) levels of expression of *HOXA2*. Survival curves were generated with Kaplan-Meier Plotter online database. See Supplementary Table S14 for complementary data. Affymetrix HOXA2 ID: 214457_at. AUC area under the curve; BC breast cancer; DMFS distant metastasis-free survival; HR hazard ratio; METABRIC Molecular Taxonomy of Breast Cancer International Consortium; N lymph-node status; OS overall survival; Pr1 probe set 1; Pr2 Probe set 2; RFS relapse free-survival; ROC receiver operating characteristic; T tumor status; TCGA the cancer genome atlas.

We assessed the diagnostic performance of *HOXA2* in BC by plotting receiver operating characteristic (ROC) curves and calculating areas under the curve (AUC). *HOXA2* expression appeared as a diagnostic indicator of BC at the immunohistochemical level in our cohort of samples (AUC = 0.99), as well as at the mRNA level in TCGA (AUC = 0.95) and the METABRIC database (AUC = 0.76 and 0.74) (Fig. 3a–d). Similarly, we observed high AUC values for *HOXA2* methylation in both the discovery and TCGA cohorts (Supplementary Fig. S1i, j).

Subsequently, we analyzed the prognostic value of HOXA2. In particular, we evaluated the expression of HOXA2 by immunohistochemistry (IHC) in 96 human BC tissue samples presenting different grading (G), tumor (T), and lymph node (N) status, according to the TNM classification (Fig. 3e, f). Multivariate analysis revealed a strong negative correlation between HOXA2 expression and G (*p* < 0.0001), T (*p* < 0.0001), and N (p = 0.0002) status according to the Pearson’s χ^2^ test (Fig. 3e, f and Supplementary Table S13). Indeed, BC patients with early-stage tumors (i.e., N0, T1, and G1) expressed higher levels of HOXA2 than advanced tumors.

Considering the prognostic interest of HOXA2, we investigated the effect of *HOXA2* mRNA expression on overall survival (OS) and relapse-free survival (RFS), as well as on distant metastasis-free survival (DMFS), of BC patients using the Kaplan-Meier Plotter (KMplot) web-based tool (kmplot.com) by merging data from different datasets (mainly GEO) (Fig. 3g–i, Supplementary Table S14). High expression of *HOXA2* denoted a favorable OS (*n* = 1 402; hazard ratio = 0.79; *p* = 0.046), RFS (*n* = 3 951; hazard ratio = 0.87; *p* = 0.02) and DMFS (*n* = 1746; hazard ratio = 0.76; *p* = 0.0062) in BC patients (Fig. 3g–i).

We conclude that high HOXA2 expression is associated with BC diagnosis, decreases with tumor progression, and indicates good prognosis.

### *HOXA2* functions as tumor suppressor in breast cancer

To investigate the role of *HOXA2* in breast carcinogenesis, the abundance of *HOXA2* was monitored in a variety of human breast cell lines by RT-qPCR. *HOXA2* was significantly downregulated in cancerous as compared to non-malignant cell lines (Supplementary Fig. S2a).

**Fig. 4:**
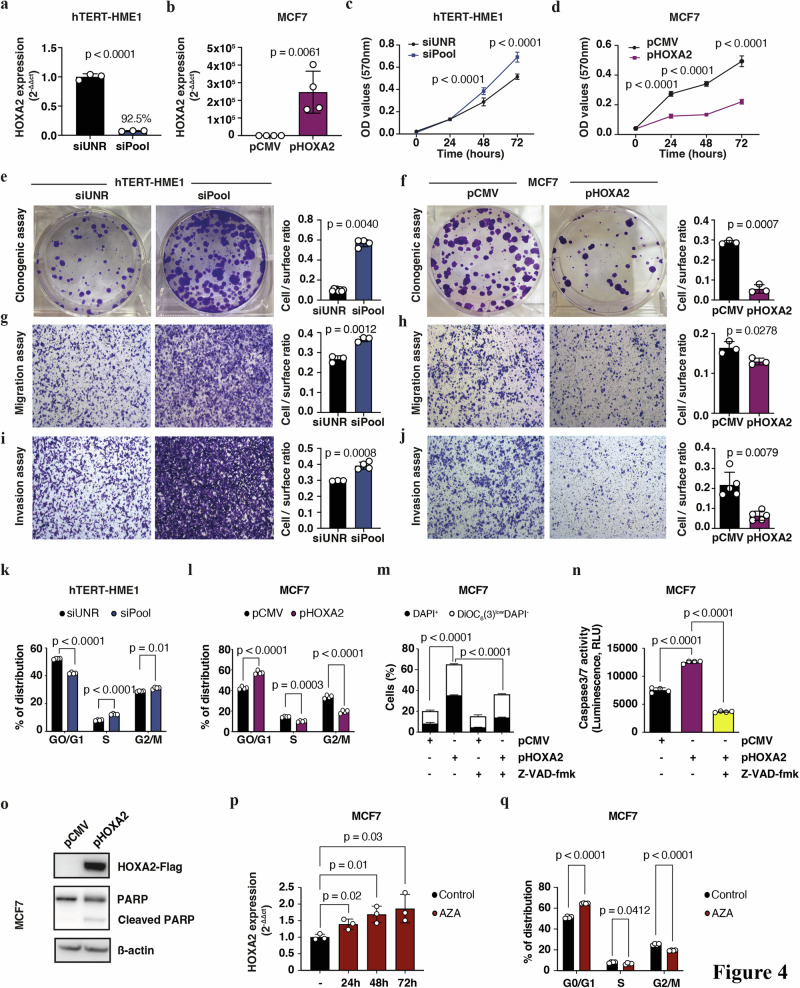
*HOXA2* functions as tumor suppressor in brs. *HOXA2* mRNA expression was measured by RT-qPCR in **a** hTERT-HME1 cells transfected with a pool of three *HOXA2*-specific siRNA sequences (siPool) or the control siRNA (siUNR), and in **b** MCF7 cells transfected with *HOXA2* plasmid (pHOXA2) or the control vector (pCMV). *GAPDH* was used as endogenous control; **a** % of knockdown (92.5%) was calculated as = (1−ΔΔCt) x100. Cell proliferation was evaluated by MTT assay in **c** hTERT-HME1 and **d** MCF7 cells following *HOXA2* silencing (**c**) or *HOXA2* forced expression (**d**). Cell proliferation after the silencing (**e**) or overexpression (**f**) of *HOXA2* in breast cell lines was measured by clonogenic assay. Cell migration was detected by transwell assay in absence (**g**) or presence (**h**) of *HOXA2* in hTERT-HME1 or MCF7, respectively. Cell invasion after *HOXA2* knockdown (**i**) or *HOXA2* overexpression (**j**) was detected by transwell assay. **e**-**j** Left panels, representative images of colonies, and transwell inserts stained with crystal violet. Right panels, corresponding quantification of colony formation, and migration and invasion efficiency. Evaluation of cell cycle perturbation by flow cytometry in **k** synchronized hTERT-HME1 cells transfected with siUNR or *HOXA2*-siPool, and in **l** synchronized MCF7 cells transfected with pCMV or pHOXA2 vectors, stained with Hoechst 33342 (10 μM). **m** Effect of the overexpression of *HOXA2* on cell apoptosis by flow cytometry in MCF7 cells transfected with pCMV or pHOXA2 plasmids in the presence or absence of the pan-caspase inhibitor Z-VAD-fmk subjected to a double staining with DAPI and DiOC_6_(3) for the detection of dying (DiOC_6_(3)^low^DAPI^−^) and dead (DAPI^+^) cells. **n** Quantification of caspase 3/7 activity in MCF7 cells transfected with pCMV or pHOXA2 in the presence or absence of the pan-caspase Z-VAD-fmk by luminescence. **o** Western blot analysis of the apoptosis-related protein PARP in MCF7 cells transfected with pCMV or pHOXA2. Antibodies for Flag or β-actin were employed as control of overexpression of HOXA2 or as loading control, respectively. **p** Bar graph representing the *HOXA2* mRNA expression in MCF7 cells after treatment with 5 μM of azacytidine (AZA) at different time points. mRNA expression was measured by RT-qPCR, normalized using *GAPDH* as endogenous control and calculated according to the 2^−∆∆Ct^ method. **q** Bar graph showing the percentage of distribution of synchronized MCF7 cells in each phase of the cell cycle in control condition and after treatment for 72 h with AZA (5 μM). **a**–**q** Data represent means ± SD from one representative experiment. Statistical significance was assessed by Student’s *t*, or by one/two-way ANOVA. AZA azacytidine; RLU relative light units; RT-qPCR reverse-transcription quantitative real time PCR; siUNR unrelated siRNA.

For functional investigations, *HOXA2* was silenced in the non-malignant human mammary epithelial hTERT-HME1 cells using three specific siRNA sequences, either in combination or alone (Fig. 4a, Supplementary Fig. S2b). The silencing strategy relying on a pool of siRNAs was particularly efficient at extinguishing *HOXA2* expression, as compared with transfections with each individual siRNA (Supplementary Fig. S2b–f) [41]. Conversely, we overexpressed *HOXA2* in malignant MCF7 cells (Fig. 4b). Next, we measured the effect of *HOXA2* dysregulation on the viability and proliferation of breast cells by means of MTT (Fig. 4c, d) and colony formation assays (Fig. 4e, f). Knockdown of *HOXA2* increased proliferation of hTERT-HME1 cells, whereas transgene-enforced overexpression of *HOXA2* reduced proliferation of MCF7 cells (Fig. 4c-f, Supplementary Fig. S2c, d). Similar results were observed in the T47D BC cell line (Supplementary Fig. S3a, b). *HOXA2* knockdown also enhanced migration and invasion, as determined in transwell assay (Fig. 4g, i, Supplementary Fig. S2e, f). Conversely, *HOXA2* overexpression reduced migration and invasion (Fig. 4h, j). *HOXA2* knockdown promoted cell cycle progression with a significant increase of the number of cells in the S and G2/M phases, as determined by flow cytometry (Fig. 4k). Conversely, the overexpression of *HOXA2* arrested cell cycle progression at the G_1_-S transition, as showed by a significant increase in the G1 phase and a decrease of cells in the S phase (Fig. 4l). The overexpression of *HOXA2* also increased the percentage of dead cells, in both the MCF7 and T47D cell lines (Fig. 4m, Supplementary Fig. S3c). This effect was dependent on caspase-induced apoptosis. Indeed, treatment with the pan-caspase inhibitor Z-VAD-fmk significantly inhibited the death of *HOXA2-*overexpressing cells (Fig. 4m) by suppressing the activity of caspases (8, 9 and 3/7) (Fig. 4n, Supplementary Fig. S3d, e). In line with this observation, *HOXA2*-overexpressing BC cells contained higher levels of the caspase-cleaved form of poly (ADP-ribose) polymerase (PARP) than control cells (Fig. 4o). Altogether, these data indicate that HOXA2 controls BC proliferation by blocking cell cycle progression and activating the apoptotic pathway.

Next, the methylation level of HOXA2 was measured in several BC cell lines *via* DNA methylation array, as well as in silico after extracting data from the CCLE database (Supplementary Fig. S3f, g). Treatment of MCF7 cells with the DNA demethylating agent 5-aza-2’-deoxycytidine (AZA) restored the expression of *HOXA2* in a time-dependent fashion (Fig. 4p). The AZA-mediated enhancement of *HOXA2* expression was associated with a partial cell cycle arrest (Fig. 4q). This observation suggests that DNA hypermethylation contributes to the transcriptional repression of *HOXA2*, thus subverting its function as a tumor suppressor.

**Fig. 5:**
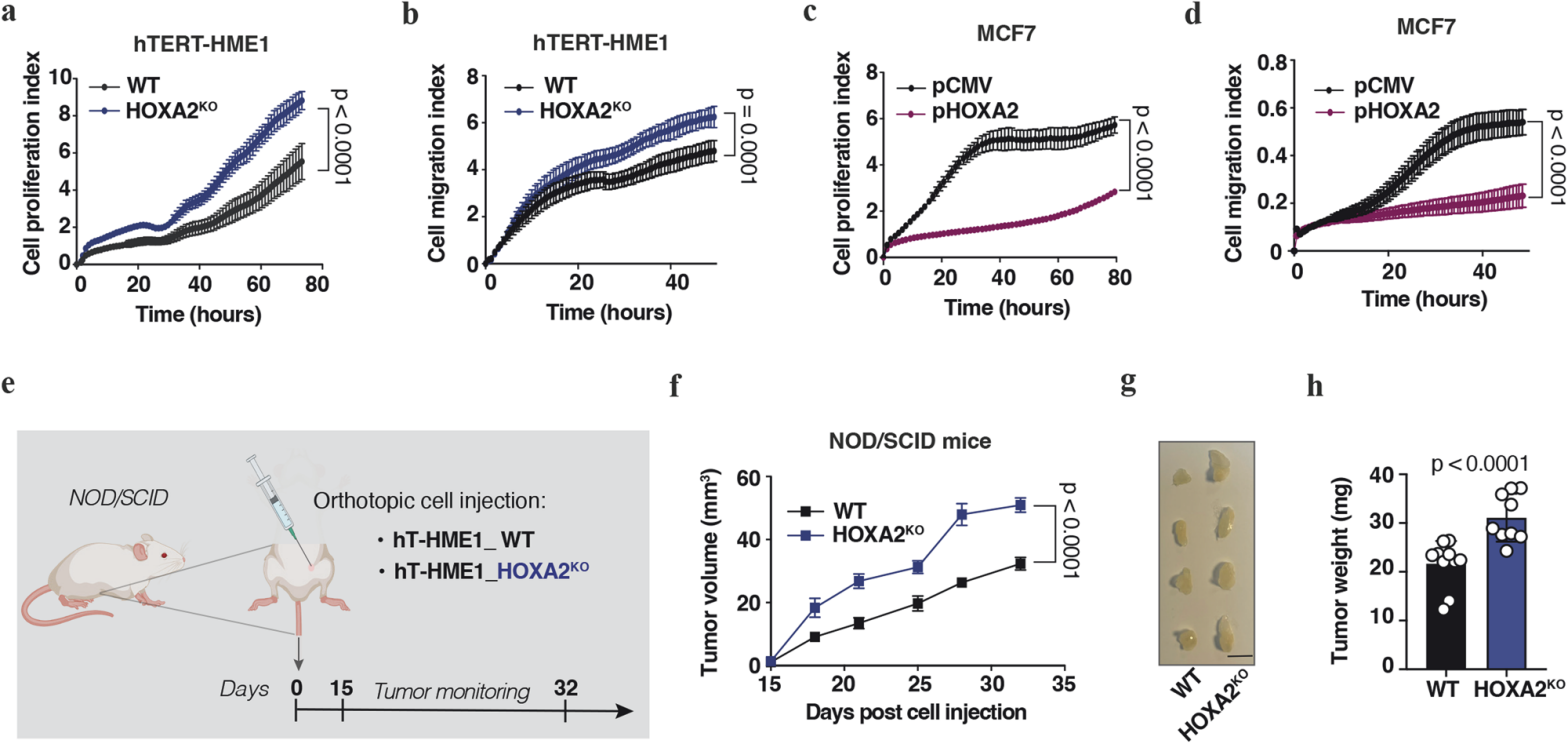
Depletion of *HOXA2* enhances breast tumor growth. Cell proliferation (**a**, **c**) and migration (**b**, **d**) indexes of hTERT-HME1 HOXA2 knockout (*HOXA2*^*KO*^) or wild type (WT) cells (**a**, **b**), and of MCF7 cells transfected with pHOXA2 or pCMV (**c**, **d**), measured in real-time by xCELLigence^®^ system. **a**–**d** Graphs show mean ± SEM from one representative experiment. Statistical significance was assessed by Student’s *t-*test. **e**–**h** Workflow of in vivo experimentation showing that hTERT-HME1 WT and *HOXA2*^*KO*^ cells were injected subcutaneously into the 4th abdominal mammary fat pad of NOD/SCID mice, and the development of the tumors was monitored over time (**e**: created with BioRender.com) (**e**). Tumor growth (**f**), size (**g**), and weight (**h**) at endpoint (*n* = 9/10 per group, mean ± SEM). **f** For comparing tumor growth curves, tumor growth *p*-values were calculated by means of a linear mixed-effect model modeling on the https://kroemerlab.shinyapps.io/TumGrowth/ platform. **g** Four representative images per group of dissected tumors are shown. Scale bar 1 cm. HOXA2^KO^, HOXA2 knockout cell line; WT wild type.

For longitudinal in vitro analysis and in vivo investigation, we stably knockout HOXA2 (*HOXA2*^*KO*^) in hTERT-HME1 cells by means of the CRISPR/Cas9 system (Supplementary Fig. S3h). Then, proliferation and migration of the *HOXA2*^*KO*^ cells were monitored in real-time using the xCELLigence® system. Consistent with previous results on *HOXA2* knockdown, *HOXA2*^*KO*^ cells displayed increased proliferative and migratory capacities (Fig. 5a, b). *HOXA2* overexpression induced the opposite effects (Fig. 5c, d).

Finally, we confirmed the oncosuppressive effect of *HOXA2* on breast tumorigenesis in vivo. For this, we injected hTERT-HME1 WT and *HOXA2*^*KO*^ cells subcutaneously into the 4th mammary fat pad of female NOD/SCID mice (Fig. 5e). The absence of *HOXA2* in cancer cells significantly accelerated tumor growth compared to the control group (Fig. 5f–h).

In sum, *HOXA2* acts as a tumor suppressor gene that controls BC progression.

### In silico prediction of *HOXA2* function in breast cancer

To investigate the biological processes and pathways related to *HOXA2*, we extracted the transcriptomic profile of BC tissues from the TCGA database (*n* = 1118), and compared the samples harboring the lowest expression of *HOXA2* (i.e., bottom quartile of tumor samples in Fig. 2i; *n* = 299) versus the specimens with the highest abundance of this gene (i.e., top quartile of tumor samples in Fig. 2i; *n* = 278). One hundred and sixty genes were differentially expressed (|log2FC| ≥ 2; *p* < 0.05) between *HOXA2* high versus low samples, with 136 genes up-regulated and 24 down-regulated (Supplementary Table S15). Next, the list of differentially expressed genes was submitted to GO, KEGG, and REACTOME enrichment analyses. Out of the 160 differentially expressed genes, GO analysis uncovered a significant enrichment (*p* < 0.05) of 43 ontology terms, of which 24 belonged to the subcategory “biological process” (BP), 11 to “cellular component” (CC), and 8 to “molecular function” (MF) (Supplementary Table S16). KEGG and REACTOME enrichment analyses highlighted an enrichment of 6 pathways (*n* = 4 for KEGG, *n* = 2 for REACTOME; Supplementary Table S17).

Across analyses*, HOXA2* dysregulation in BC tumors coincided with variations in genes related to tissue development, including decreased expression of other *HOXA* genes (i.e., *HOXA3*-*7*, and *9*). At the cell scale, processes referring to cell-cell and cell-extracellular milieu interactions were affected, with a particular enrichment of genes associated with ligand-receptor binding and synaptic transmission. Notably, the expression of genes encoding neuroactive molecules or related receptors was modulated, including an upregulation of *TAC1* (tachykinin precursor 1), *EDN3* (endothelin 3), *PENK* (proenkephalin), *GRIA4* (Glutamate receptor ionotropic, AMPA 4) and *HTR2A* (5-hydroxytryptamine receptor 2 A), and a down-regulation of *GRIA1*, *TRH* (thyrotropin-releasing hormone), or again *CHRNA9* (cholinergic receptor nicotinic alpha 9 subunit). At the intracellular level, peroxisome proliferator-activated receptor (PPAR) activity and lipid metabolism appeared impacted by the loss or decrease of *HOXA2* expression. This latter observation was illustrated by the down-expression of genes encoding for aquaporin-7 (*AQP7*), fatty acid-binding protein 4 (*FABP4*), perilipin 1 and 4 (*PLIN1*, *PLIN4*), and cell death-inducing DFF-like effector A and C (*CIDEA*/*CIDEC*), hydroxysteroid 17β-dehydrogenase 13 (*HSD17B13*), or related hormones like leptin (LEP) and adiponectin (*ADIPOQ*) in *HOXA2* low samples (Supplementary Tables S16 and S17). Of note, most of these genes showed a positive regulation upon loss or decreased expression of *HOXA2* in BC, with the exception of the genes *GRIA1*, *thyrotropin releasing hormone (TRH)*, *Cholinergic Receptor Nicotinic Alpha 9 Subunit (CHRNA9)*, *Wilms’ tumor gene 1* (*WT1*), and *slit guidance ligand 1* (*SLIT1*) (Supplementary Table S17).

These observations were further validated when comparing the transcriptomic profile of hTERT-HME1 cell lines WT or KO for *HOXA2*. More precisely, we observed a HOXA2-dependent differential expression of genes related to lipid metabolism (Supplementary Tables S18—see KEGG terms in bold, including “regulation of lipolysis in adipocytes”). Additional pathways were affected upon disruption of HOXA2 and involved in other metabolic signaling (e.g., amino acids), cell proliferation and adhesion, cell-cell interactions, and immune activity (Supplementary Tables S18).

Collectively, these results suggest that the deregulation of *HOXA2* interferes with intracellular signaling pathways and surface sensors that could influence the progression and aggressiveness of BC.

### HOXA2 enhances the expression of PPARγ and CIDEC

**Fig. 6:**
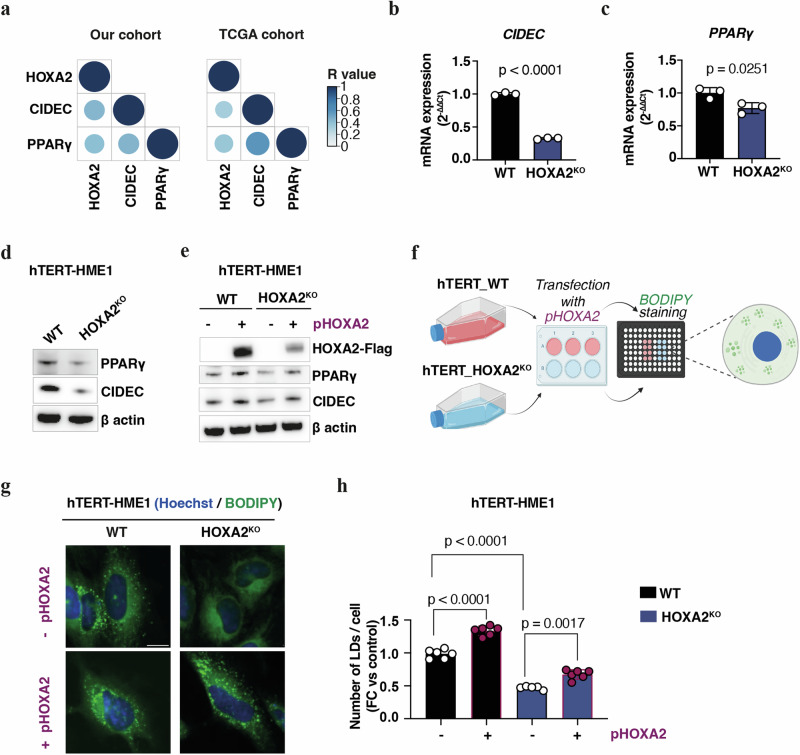
HOXA2 abundance modulates the expression of CIDEC and PPARγ in lipid metabolism of BC. (**a**) Bubble plots showing the significant positive correlation values (R∈[0;1], p-value ≤ 0.05) between the expression of the three selected genes (*HOXA2*, *CIDEC* and *PPARγ)* in breast non-tumor/normal and cancer tissues from our cohort (left graph) and from TCGA dataset (right graph). Bubble color (from light to dark blue) indicates the strength (R value) of the correlation. Of note, a strong correlation is in dark blue and corresponds to a R = 1, while a weak correlation is in light blue and corresponds to a R = 0. Bubble size indicates the significance (p-value) of the correlation; bigger is the size of the bubble, lower is the p-value. (**b,** **c**) The mRNA expression of *CIDEC* (**b**) and of *PPARγ* (**c**) was measured in hTERT-HME1 cells wild type (WT) or knockout (*HOXA*^*KO*^) for *HOXA2* by RT-qPCR. *GAPDH* was used as endogenous control. (**d,** **e**) Western blot analysis of the expression of CIDEC and PPARγ in WT and *HOXA2*^*KO*^ cells (**d**) and in WT and *HOXA2*^*KO*^ cells transfected with *HOXA2* plasmid (pHOXA2) or the control vector (pCMV) (**e**). Antibodies for Flag (**e**) or β-actin (**d,** **e**) were employed as control of overexpression of *HOXA2* or as loading control, respectively. Experimental workflow employed for lipid droplet tracing (**f**) (**f**; created with BioRender.com). Confocal microscopy images (scale bar equals 10 μm) (**g**) and quantification of the number (**h**) of intracellular lipid droplets (LDs) normalized to cell area and expressed as fold change (FC) to control in hTERT-HME1 WT and *HOXA2*^*KO*^ cells with (+pHOXA2) or without (−pHOXA2) restoration of HOXA2 expression. LDs were stained with BODIPY 493/503 (green), and nuclei were stained with Hoechst (blue). **b**–**e**, **h** Data are presented as mean of ± SD from one representative experiment and analyzed with Student’s *t*-test or ANOVA. FC fold change; hTERT_WT hTERT-HME1 wild type cells; hTERT_HOXA2^KO^ hTERT-HME1 HOXA2 knockout cells; RT-qPCR reverse-transcription quantitative real time PCR.

Next, we wondered whether *HOXA2* might regulate the expression of genes involved in lipid metabolism (e.g., *ADIPOQ*, *AQP7*, *PLIN1*), and in the PPAR signaling pathway (e.g., *CIDEC*, *FABP4*) (Supplementary Table S17). *HOXA2* demonstrated a significant positive correlation with the expression of *CIDEC* (also known as fat-specific protein 27, *FSP27*) and of *PPARγ*, in both our cohort of BC samples (*HOXA2/CIDEC*: R = 0.43, *p*-value = 0.001; *HOXA2/PPAR*γ: *R* = 0.4, *p*-value = 0.003) and in TCGA database (*HOXA2/CIDEC*: R = 0.3, *p*-value = 1.24e-26; *HOXA2/PPAR*γ: *R* = 0.36, *p*-value = 3.3e-35) (Fig. 6a). *CIDEC* and *PPAR*γ strongly correlated among each other in both BC datasets (our cohort: *R* = 0.44, *p*-value = 0.0007; TCGA data: *R* = 0.6, *p*-value = 8e-100) (Fig. 6a). These data were also supported by the transcriptomic analysis performed on the human breast cells subjected to the knockout for *HOXA2*. Notably, the suppression of HOXA2 led to the decrease of mRNA expression of *CIDEC* (FC = −9.8, *p*-value = 4.23e-05) and *PPAR*γ (FC = −1.7, *p*-value = 0.01) (Supplementary Table S19). Of note, *CIDEC* was previously reported as a direct target of PPARγ in adipocytes, as well as in the liver of obese mice [42, 43]. Based on these observations, we decided to further investigate the potential interplay between HOXA2 and PPARγ and CIDEC.

We measured the abundance of CIDEC and PPARγ in hTERT-HME1 cells WT and KO for *HOXA2* by RT-qPCR and immunoblotting. Consistent with the transcriptomic data, we found that the expression levels of CIDEC and PPARγ decreased upon HOXA2 depletion at the mRNA and protein levels (Fig. 6b–d). We then corroborated this correlation by rescuing *HOXA2* expression in *HOXA2*^*KO*^ cells. Transfection-enforced re-expression of *HOXA2* effectively restored the protein levels of PPARγ and CIDEC in *HOXA2*^*KO*^ cells, reaching those found in WT cells (Fig. 6e). These results plead in favor of the capacity of HOXA2 to upregulate the expression of PPARγ, which then transactivates CIDEC.

It is well known that CIDEC plays a critical role in the formation and size of lipid droplets (LDs) in adipocytes (mainly), both in vitro and in vivo [44]. Accordingly, we observed a marked reduction of the number of BODIPY 493/503-stained lipid droplets in hTER-HME1 cells following *HOXA2* depletion as compared to control cells (Fig. 6f–h). In addition, this decrease in lipid levels was reversed after re-expression of *HOXA2* in *HOXA2*^*KO*^ cells (Fig. 6g, h), confirming the implication of HOXA2 in lipid metabolism.

Altogether, these results demonstrated that the absence of HOXA2 reduces the expression of PPARγ and CIDEC, and in turn lipid storage in BC cells. This supports a role for HOXA2 in breast carcinogenesis-relevant energy metabolism.

## Discussion

Biomarkers are significantly improving the management of BC [45]. Indeed, histological assessment of ER, PR, Ki67 and HER2, along with multigene tests (e.g., PAM50), are used in the routine management of BC [1]. The evaluation of altered (epi)genetic mechanisms, including DNA methylation affecting the expression of genes, pseudogenes and non-coding RNAs is providing additional insights for the diagnosis, prognostication and personalized treatment of BC [14, 46, 47]. In this context, we applied an integrative analysis of DNA methylation and RNA-sequencing, followed by in-depth bioinformatics analyses, to identify novel tumor suppressor genes. Amongst the genes that were altered at the transcription and epigenetic levels, we focused on *HOXA2*, taking into consideration that epigenetic events, often hyper/hypo-methylation, can contribute to aberrant expression of *HOX* genes (e.g., *HOXA1, HOXA5*, and *HOXA9*) in BC [19, 21, 22]. Moreover, altered abundance and methylation status of *HOXA2* have been proposed as prognostic indicators in a variety of cancers (e.g., lung SCC, CRC, and prostate cancer) [28–33], but had never been studied in BC.

Here, we report that *HOXA2* acts as a tumor suppressor in BC. As compared to normal breast tissue, *HOXA2* expression was significantly reduced in malignant specimens (along with an increased methylation status) in both our discovery cohort and validation datasets (TCGA and METABRIC). In addition, in our cohort of BC patients and in the TCGA dataset, *HOXA2* exhibited a significant negative correlation between its levels of expression and its methylation status. This suggests a strong link between *HOXA2* downregulation and breast carcinogenesis. Such diagnostic potential of *HOXA2* in BC was confirmed by ROC/AUC calculations. Interestingly, HOXA2 downregulation was accentuated with tumor progression, meaning that it was more pronounced in high-grade, advanced-stage, and metastatic BC, as determined by IHC. Accordingly, low expression of *HOXA2* correlated with unfavorable outcomes.

In vitro and in vivo experiments support the tumor suppressor properties of *HOXA2* in BC. Consistent with the results obtained on tissue specimens, *HOXA2* showed a higher level of expression in normal cells than in BC cells. In addition, *HOXA2* knockdown and knockout promoted proliferation and drove invasion and migration. Vice versa, upregulation of *HOXA2* by transfection of the gene or by inhibition of DNA methylation reduced tumor cell viability, migration, and invasion. Moreover, enhanced expression of *HOXA2* in BC cells triggered apoptosis, as indicated by high activity of pro-apoptotic caspases. Additionally, BC tumor xenografts lacking *HOXA2* appeared more aggressive in mice. These results describe a tumor-suppressive function of HOXA2 in the breast that diverges from its pro-oncogenic activity reported in prostate cancer, lung SCC, and CRC. [28–33] Future investigations must determine which tissue or cell type-specific factors may explain the pro-tumorigenic versus tumor-suppressive effects of *HOXA2* in distinct organs.

In BC, we demonstrated that the altered expression of *HOXA2* results in an unbalanced lipid metabolism by decreasing the levels of PPARγ and CIDEC. These functional insights were gained through in silico investigations and validated in vitro. Initially, we performed a comparative analysis of the transcriptomic profiles of BC tissue samples with the highest versus lowest expression of *HOXA2*. Subsequently, gene ontology annotation and enrichment analyses were performed to elucidate the biological functions of these genes. These analyses revealed a spectrum of genes and pathways affected by the loss of *HOXA2*. Notably, they were predominantly associated with PPAR signaling pathway (e.g., *ADIPOQ*, *PCK1*), as well as with lipid content regulation, including fatty acid metabolism (e.g., *PLIN1*, *PLIN4*), fatty acid transport (e.g., *FABP4*, *AQP7*), and lipid storage (e.g., *CIDEC*).

In our enrichment analysis, the adipocytokine/PPAR signaling pathway was overrepresented. This pathway regulates several cellular and physiological processes, including cell proliferation and apoptosis, glucose and lipid metabolisms, or again inflammation [48]. Similarly, KEGG enrichment analysis of downregulated genes in *HOXA2* knockout cells as compared to WT cells, confirmed that lipid storage metabolism was significantly affected by the depletion of *HOXA2*. Along this line, Marino and colleagues recently evidenced an upregulation of the lipid metabolism and genes involved in adipogenesis in the breast prior to the clinical manifestation of malignancy [49]. Among the genes identified in the adipocytokine/PPAR signaling pathway, *CIDEC* and *PPARγ* were positively correlated with the expression of *HOXA2*. Accordingly, depletion of *HOXA2* led to low mRNA and protein levels of PPARγ and CIDEC in BC cells. Our findings are consistent with previous evidence reporting PPARγ as a transcriptional regulator of CIDEC in adipocytes [42, 43]. Along this line, it has been reported that decreased levels of the tumor suppressor PPARγ correlate with unfavorable prognosis in BC [50]. Furthermore, preclinical studies showed that the activation of PPARγ, using natural or synthetic agonizts, represents a promising approach for the treatment of BC [51]. *CIDEC*, also known as *FSP27*, is a master regulator of lipid metabolism [44]. Specifically, it is an LD-associated protein, which is required for the formation of unilocular LDs in adipocytes [44]. Depletion of *CIDEC* in 3T3-L1 adipocytes decreased cytoplasmic LDs and promoted triglyceride accumulation [52]^.^ Similarly, we found that the ablation of *HOXA2* expression significantly reduced LD content in BC cells. Remarkably, rescuing *HOXA2* expression in *HOXA2* knockout cells, restored the abundance of LDs, thereby supporting the involvement of *HOXA2* in the imbalanced lipid metabolism occurring during breast cancer development/progression. In line with our results, the transformation of normal breast tissue to invasive carcinoma is accompanied with a decrease of cytoplasmic LDs [53]^.^ Moreover, a recent research by Wright and collaborators demonstrated that the *CUB-domain containing protein 1* (*CDCP1*) gene contributes to TNBC metastasis by reducing LDs [54]. Further investigations will shed light on whether HOXA2 deregulation influences the crosstalk between tumor and adjacent mammary tissues, such as the adipose tissue, in a way that favors breast oncogenesis [55].

In summary, this work reveals *HOXA2* as a novel tumor suppressor gene controlling breast tissue carcinogenesis. Our results unravel a link between local HOXA2 downregulation and breast carcinogenesis. This downexpression correlates with a specific DNA hypermethylation. Furthermore, decreased levels of HOXA2 were associated with advanced BC and unfavorable clinical outcomes. Extensive annotation and enrichment analyses, corroborated by in vitro investigations, revealed the interplay between HOXA2 and some genes involved in lipid metabolism, including PPARγ and CIDEC. These results suggest that alterations in lipid metabolism affect breast carcinogenesis. In the perspective of a clinical application for the diagnosis and prognosis of BC patients, it will be of interest to study whether the hypermethylation of *HOXA2* can be detected in the tumor circulome (i.e., circulating tumor cells), or tumor-derived organelles and cell-free DNA. Moreover, the pathway linking HOXA2 and lipid metabolism- might constitute target(s) for developing novel therapeutic approaches against BC.

## Materials and methods

### Human sample collection and processing

Tissue samples (*n* = 12 normal/non-tumor and  *n* = 31 BC specimens) were collected from the patients attending the BC Department of the “Istituto Nazionale dei Tumori - Fondazione G. Pascale” of Naples, Italy. After collection, tissue specimens were immediately cryopreserved until processing as described previously [14]. Histological and molecular tests were performed to evaluate the immunoprofile of each breast tissue sample.

### Cell lines and culture conditions

Human breast cancer cell lines (MCF7, T47D, and BT549) and the immortalized normal human mammary epithelial cell line (hTERT-HME1) were used. hTERT-HME1 cells were cultured in DMEM/F12 supplemented with epidermal growth factor (EGF) 20 ng/ml, insulin 10 μg/ml, hydrocortisone 0.5 μg/ml, and 10% fetal bovine serum (FBS) and 1% penicillin–streptomycin. MCF7 cells were cultured in Eagle’s minimum essential medium (ATCC #30-2003) supplemented with 2 mM glutamine, 1% non-essential amino acids, 1% penicillin–streptomycin, and 10% FBS. T47D and BT549 cells were cultured in DMEM and RMPI-1640 medium complemented with 1% penicillin–streptomycin and 10% FBS, respectively. Cells were cultured in a humidified 5% CO_2_ atmosphere at 37 °C. Cell lines tested were negative for mycoplasma contamination and were passaged < 10 times after the initial revival from frozen stocks. All reagents (when not specified) are from Sigma-Aldrich. The culture media and supplements for cell culture were purchased from Gibco-Life Technologies^TM^ and plasticware from Corning Inc. unless otherwise specified. hTERT-HME1 cell line was kindly provided by Dr. Federica Di Nicolantonio (University of Turin, Italy), and BC cells were supplied by the cell culture facility of CEINGE- Biotecnologie Avanzate Franco Salvatore (Naples, Italy).

### Transfection conditions

For *HOXA2* knockdown, hTERT-HME1 cells were transfected with either 3 single specific *HOXA2*-siRNAs (siR1: #s6755, siR2: #s223861, Thermo Fisher Scientific (Waltham, MA, USA); siR3: #SI04186609, QIAGEN) and the pool of the 3 siRNAs (siPool), or with a non-targeting siRNA (siUNR) (#SIC001, Sigma-Aldrich) using Lipofectamine® RNAiMAX reagent (Invitrogen^TM^), according to the manufacturer’s instructions. To establish HOXA2 overexpressing cells,, cell lines (e.g., MCF7, T47D) were transfected with either *HOXA2* Human FLAG vector or control pCMV empty vector [56] using Viafect® Transfection Reagent (Promega), as recommended by the manufacturer.

### Generation of stable *HOXA2* knockout cell line

The *HOXA2* knockout (*HOXA2*^KO^) stable cell line was generated by means of a CRISPR/Cas9 system. hTERT-HME1 cells were transfected with two synthetic *HOXA2* RNA guides (gRNAs) (gRNA1: #CRISPR1064084_SGM, gRNA2: #CRISPR487239_SGM, from Thermo Fisher Scientific) using lipofectamine CRISPRMAX^TM^ reagent and Cas9 nuclease protein (Invitrogen^TM^), according to the manufacturer’s protocol. After 48 h, cells were collected and sorted to select single-cell clones using BD FACSAria flow cytometer. *HOXA2*^KO^ cell line was then subjected to RNA-sequencing as described below. The knockout efficiency of selected *HOXA*^*KO*^ clone was verified by RT-qPCR, as well as by RNA-sequencing.

### RNA and DNA extraction

Total RNA and genomic DNA were co-isolated from microdissected tissues (31 tumors, 12 non-tumor/normal) using the phenol/guanidine-based QIAzol Lysis Reagent method (Qiagen). RNA was isolated from the aqueous phase, while DNA from the interphase and organic phase, according to the manufacturer’s instructions. RNA was subsequently purified using the RNeasy Mini kit (Qiagen). RNA from cell lines, including *HOXA2*^*KO*^ cell line, was extracted using the RNeasy Plus kit (Qiagen). The quantity and purity of RNA and DNA were measured using the NanoDrop™ spectrophotometer (Thermo Fisher Scientific).

### High-throughput sequencing

Transcriptomic assay on RNA samples derived from microdissected tissues (*n* = 43) was carried out on Illumina HiSeq platform according to Illumina protocol, as described previously [14]. Briefly, RNA sequencing libraries were prepared using TruSeq Stranded Total RNA samples with Ribo-Zero Gold rRNA removal kit (Illumina, San Diego, CA, USA). Sequencing was performed on a HiSeq 1500 platform (Illumina). Messenger RNA-sequencing (mRNA-Seq) was also performed on hTERT-HME1 *HOXA2*^*KO*^ and WT cells (*n* = 4 biological replicates/condition). Library preparation and mRNA-seq were performed by Novogene.

### Infinium methylation array

Genomic DNA was bisulfite converted using the EZ DNA Methylation kit (ZYMO Research), according to manufacturer’s instructions. A 100%-methylated control was used as positive control to assess bisulfite conversion efficiency (#D5011, ZYMO Research). DNA methylation levels were assessed through the Illumina Infinium HumanMethylation450 BeadChip array (Illumina), which covers approximately 450.000 CpG sites. BeadChips were imaged with the Illumina iScan system.

### RT-qPCR

First, 1 μg of total RNA was reverse transcribed using Superscript IV VILO Master MIX (Invitrogen^TM^). Then, 100 ng of cDNA were amplified with specific Taqman probes (*HOXA2:* Hs00534579_m1, *PPARG:* Hs01115513_m1, *CIDEC*: Hs01032998_m1, all from Thermo Fisher Scientific) carrying out the qPCR on the StepOne^TM^ Real-time PCR system (Thermo Fisher Scientific) using TaqMan Fast Advanced Master Mix (Applied Biosystems^TM^), according to the manufacturer’s instructions. Relative expression was calculated according to the 2^−∆∆Ct^ method, normalizing mRNA expression levels to *GAPDH* (#4333764, Thermo Fisher Scientific), the endogenous control.

### Immunohistochemistry (IHC)

HOXA2 protein expression was determined on two commercially available breast tissue microarrays (#BRC1021 and #BRC1501, US Biomax). Ninety-six selected cases of human breast cancer tissues with different grading and classification of malignant tumors (TNM) status were stained using a rabbit polyclonal anti-HOXA2 antibody (1:200, #HPA029774, Sigma-Aldrich) and analyzed by light microscopy. The percentage of HOXA2-positive stained cells in the cytoplasm was estimated in at least 5 significant fields and in more than 500 cells, and scored by 2 pathologists as follows: score 1 = less than 1% of positive cells (absent to low expression of HOXA2); score 2 = from 1% to 20% of positive cells (medium expression of HOXA2); score 3 = more than 20% of positive cells (high expression of HOXA2).

### MTT assay

Cells were seeded at a density of 2000 (hTERT-HME1) and 3000 (MCF7, T47D) cells/well in 96-well plates and transfected with *HOXA2*-targeting siRNAs or a *HOXA2*-encoding plasmid. Scrambled siUNR and pCMV plasmid served as controls. After the indicated time points, cells were incubated with 10 μl of 3-(4,5-dimethyl-2-thiazolyl)-2,5-diphenyl- 2H-tetrazolium bromide (MTT) (Sigma-Aldrich, 5 mg/mL) at 37 °C for 4 h. Then, the medium was replaced with 100 μl of dimethylsulfoxide (DMSO, Sigma-Aldrich), incubated for 15 min and, lastly, the absorbance was measured at 570 nm using Victor X4 plate reader (PerkinElmer).

### Transwell migration and invasion assays

The impact of *HOXA2* on cell migration and invasion was performed using 24-well Transwell inserts (8 μm pore size; #PI8P01250, Corning Costar). For invasion, upper chambers of the inserts were pre-coated with 100 μl of 2 mg/mL of Matrigel® (Corning®). Briefly, cells were transfected in 6-well plates. Then, cells were trypsinized, washed twice in Phosphate-Buffer Saline (PBS), counted, and seeded at 5 × 10^4^ (hTERT-HME1) or at 8 × 10^4^ (MCF7) cells in serum-free media in the upper chamber. Complete medium with 10% FBS was used as chemoattractant in the lower chamber. After 24 h (hTERT-HME1) or 48 h (MCF7) of incubation at 37 °C, cells on the upper compartment were removed with a cotton swab, while cells retained in the insert were fixed in 5% glutaraldehyde (#G6257, Sigma-Aldrich), stained with 1% crystal violet (#C3886, Sigma-Aldrich) and washed twice in distilled water. Cells retained in the porous membrane were viewed under a microscope and five selected areas of the insert were photographed using Zeiss AxioVert200 microscope (2.5x). For quantification, the surface of the stained cells was detected for each selected area and the ratio between total cell surface and image surface was calculated. Then, results of surfaces in the view fields per replicate were combined, and the means were calculated for each condition.

### Clonogenic assay

Cells were transfected as previously described in the “Transfection” section. After the indicated time point, 500 cells/well were seeded in 6-well plates. After 2–3 weeks, the cells were fixed, stained with crystal violet, and photographed. The percentage of area covered by crystal violet-stained cell colonies was quantified by calculating the ratio between total colonies' surface and image surface.

### xCELLigence® real-time cell analysis (RTCA) system

Proliferation and migration capacity of *HOXA2*^*KO*^ cell line and *HOXA2* overexpressing cells (*HOXA2*^*OV*^) were monitored using the xCELLigence^**®**^ RTCA instrument (ACEA Biosciences, CA, USA), according to the manufacturer’s instructions. Briefly, to assess cell proliferation, cells (*HOXA2*^*KO*^: 2 × 10^3^ cells/well; *HOXA2*^*OV*^: 8 × 10^3^ cells/well) were seeded in serum-free media in E-16 plates, and cell indexes were recorded. The migration ability of *HOXA2*^*KO*^ and *HOXA2*^*OV*^ cells was monitored in CIM-16 plates (4 × 10^4^ cells/well and 3 × 10^4^ cells/well, respectively) by the RTCA. Medium with 10% of FBS was used as chemoattractant.

### Cell cycle analysis

For cell cycle distribution assessment, cells were seeded in 6-well plates and synchronized in G_1_/S by a double thymidine blocking (2 mM, Sigma-Aldrich) [57]. Then, cells were transfected for overexpression or knockdown approaches. Subsequently, cells were detached, washed with PBS, and stained with 10 μM of Hoechst 33342 (Thermo Fisher Scientific) in 600 μl of complete medium for 45 min at 37 °C, protected from light. DNA staining was measured using Attune® Ntx Flow Cytometer (Thermo Fisher Scientific).

To measure apoptotic features, cells were plated in 12-well plates at a density of 5 × 10^4^ per well and transfected with either pCMV empty vector or *HOXA2*-plasmid in absence or presence of the caspase inhibitor Z-Val-Ala-Asp fluoromethylketone (Z-VAD-fmk, 50 μM) (#S7023, SelleckChem). Then, transfected cells were collected and co-stained for 30 min at 37 °C with 1 μg/mL of 4′,6-diamidino-2- phenylindole (DAPI, Thermo Fisher Scientific), which only accumulates in cells with permeabilized plasma membranes, and with 20 nM of 3,3′-dihexiloxalocarbocyanine iodide (DiOC_6_(3)), a mitochondrial transmembrane potential‐sensitive dye, for the cytofluorimetric detection of dying (DiOC_6_(3)^low^ DAPI^-^) and dead (DAPI^+^) cells.

Cytofluorometric acquisitions were performed on Miltenyi cytofluorometer (MACSQuant® Analyzer 10). Flow cytometry analyses were carried out using the FlowJo software (LLC, Oregon, USA) upon gating on events exhibiting normal forward scatter (FSC) and side scatter (SSC) parameters.

### Caspase activity assay

Cells were transfected in 6-well plates as described before. After transfection, cells were seeded in 96-well plates at 3500 cells/well in the presence or absence of the pan-caspase inhibitor Z-VAD-fmk (50 μM, #S7023, SelleckChem) for 24 hours. Then, caspase activity was measured using the Caspase Glo kit (Caspase 3/7: #G8091, Caspase 8: #G8200, Caspase 9: #G8210, Promega), according to the manufacturer’s instructions. Luminescence was measured using Victor X4 plate reader (PerkinElmer).

### Lipid droplet (LD) staining

HTERT-HME1 WT and KO for *HOXA2* were transfected using the *HOXA2* Human FLAG vector or control pCMV as described before (see “Transfection condition”). Then, for the quantitative assessment of intracellular lipid formation, cells were plated in 96-well plates overnight. Next, cells were fixed with a mix of 4% paraformaldehyde and Hoechst (1 µg/ml) for 20 min at RT and then stained with 10 μM BODIPY 493/503 (in PBS, #D-3922, Invitrogen^TM^) for 30 min at RT, followed by washing with PBS. LDs were visualized by confocal microscopy (Molecular Device IXM-C, equipped with CFI PlanApo Lambda 20× objective, 9 view fields per well). For LD quantification, at first cell nuclei were detected using Hoechst staining, and labeled by means of a watershed algorithm. The obtained mask was then used as a seed for the Voronoi-based propagation method, allowing for cytoplasm segmentation in the LD fluorescence channel. Finally, LD “dots” were segmented after applying a top-hat filter to the original image, followed by sigmoidal transformation and binarization. Dot count and total area were calculated for each cell, normalized to cytoplasmic area, and averaged by view field.

### Immunoblotting

After treatment, cells were harvested and lysed on ice for 1 hour in a lysis buffer containing a cocktail of protease and phosphatase inhibitors as previously described [57]. Lysates were centrifuged for 30 min at 4 °C and the supernatants collected. The protein concentration of the supernatants was quantified using the Pierce^TM^ BCA protein assay (#23225, Thermo Fisher Scientific). After heat-denaturation at 95 °C for 10 min in reducing sample buffer (#NP0080, Invitrogen^TM^), proteins (20 μg) were separated by pre-cast 4–12% NuPAGE Novex Bis-Tris (Invitrogen^TM^) gel polyacrylamide electrophoresis and electrotransferred onto polyvinyl polypyrrolidone Immobilon^TM^ membranes (Millipore Corporation). Non-specific binding was blocked with 5% skimmed milk for 1 h, and membranes were probed with specific primary antibodies (anti-cleaved PARP: #9542, Cell signaling; anti-Flag: #15793, Cell signaling; anti-PPARγ: #ab178860, Abcam; anti-CIDEC: #PA1-4316, Invitrogen) followed by incubation with horseradish peroxidase-labeled secondary antibodies. β-actin (#ab20272, Abcam) served as loading control. Proteins were detected using SuperSignal West Pico chemiluminescent substrate (Thermo Fisher Scientific). Immunoblots were imaged using ImageQuant^TM^ 800 imaging system and were quantified by ImageJ.

### Demethylating treatment

Cells were treated using the demethylating agent 5-aza-2′- deoxycytidine (AZA) at a concentration of 5 μM (#A3656, Sigma-Aldrich). Untreated cells served as control. For 72 h treatments, AZA was refreshed daily.

### Mouse strains and accommodation

Six- to eight-week-old female NOD/SCID mice (*n* = 20) were purchased from Envigo (France). Mice were allowed to acclimate for at least 1 week. They were housed in a pathogen-free and temperature-controlled environment with 12 h light/dark cycles and received water and food ad libitum.

### Xenograft tumor model

hTERT-HME1 WT and *HOXA2*^*KO*^ cells were cultured as described before. The day of the injection, cells were trypsinized, washed once in complete culture medium, and then twice in culture medium without (w/o) FBS and w/o PS. For the tumor grafting, 2 × 10^6^ cells of hTERT-HME1 WT or *HOXA2*^*KO*^ suspended in 100 µl of a 1:1 solution of Corning Matrigel® Matrix high concentration (Corning) and medium (w/o FBS and w/o PS), were injected subcutaneously into the 4th abdominal mammary fat pad of female, 6–8 week-old NOD/SCID mice (*n* = 20), using a 25-gauge needle. Mice were randomized into two groups of equivalent mean tumor size 1-week post-tumor cell engraftment. Tumor growth was blindly measured two times per week starting at day 15 after tumor injection using a digital caliper. Tumor volume (mm^3^) was calculated using the following formula: π/6 × (length × width × height). Mice were euthanized by cervical dislocation when reaching endpoint (e.g., tumor size < 1500 mm^3^, ulcerated tumor, weight loss > 20%, degraded general body condition) or at the end of the experiment at day 54.

### Bioinformatics and statistical analysis

#### Transcriptomic and methylome data analysis

RNA-sequencing data of our cohort of BC patients were processed as described elsewhere [14]. Briefly, differentially expressed genes were identified using DESeq2 [58]. Only genes with *p*-value ≤ 0.05 and absolute log2 fold change (FC) ≥ 1 were considered to be differentially expressed. Differential expression of genes extracted from the mRNA-seq on hTERT-HME1 WT and HOXA2^KO^ cell lines was performed by Novogene using DESeq2 R package (1.20.0). P-values were adjusted using the Benjamini and Hochberg’s approach for controlling the false discovery rate (FDR). Genes with p-value ≤ 0.05 and absolute log2 FC  ≥ 1 were considered as differentially expressed. Concerning in silico analyses, data on breast cell line methylation values were downloaded from the Cancer Cell Line Encyclopedia (CCLE, https://portals.broadinstitute.org/ccle) database. RNA-sequencing and DNA microarray data of BC patients from The Cancer Genome Atlas (TCGA) were downloaded from the website Genomic Data Commons (https://portal.gdc.cancer.gov) using TCGA biolinks R packages [59]. Gene differential expression was evaluated using DESeq2 [58]. A gene was considered significantly differentially expressed when *p*-value was ≤ 0.05 and absolute fold change ≥ 2. Initial methylation beta-values were converted to *M*-value by minify function using the following formula: M=logit(Betavalue)=log2(Methylated/unmethylated). *M*-values were used for differential methylation analysis. Differentially methylated CpG islands were identified using the limma package [60]. Only islands with *p*-value ≤ 0.05 and an absolute fold change ≥ 1.45 were considered as differentially methylated. Wilcoxon-Mann-Whitney U tests were performed to evaluate the differential expression and methylation of HOXA2 in BC. All analyses were conducted with R (v4.0.5).

The mRNA expression of *HOXA2* in normal and malignant BC tissues was also evaluated using the Molecular Taxonomy transcriptomic of Breast Cancer International Consortium (METABRIC) dataset [61]. The two probe sets of HOXA2 transcript were used for differential expression analysis. *P*-values of differential expression have been obtained by using R-package limma [60].

### Survival curve analysis

The effect of *HOXA2* on BC patient overall survival (OS), relapse-free survival (RFS), and distant metastasis-free survival (DMFS) was analyzed using Kaplan-Meier Plotter database (https://kmplot.com), by merging data from different datasets (Gene Expression Omnibus, GEO, and ArrayExpress) [62]. The individual datasets used are listed in Supplementary Table S14. Survival curves were generated by selecting as filters “the best cutoff” and “JetSet best probe set”.

### Receiver operating characteristic (ROC) curve analysis

ROC curves were generated and area under the ROC curve (AUC) was calculated using “MASS” and “pROC” R packages [63, 64].

### Correlation analysis

Correlation analyses were performed with the “cor.test” R function. Spearman and Pearson correlation coefficients (ρ and R, respectively) and the relative p-values were calculated by “Spearman rank correlation test” and “Pearson correlation test”, respectively.

### Gene ontology and pathway enrichment analyses

Gene Ontology (GO), Kyoto Encyclopedia of Genes and Genomes (KEGG), and REACTOME enrichment analyses were executed on differentially expressed genes extracted from our clinical BC tissue RNAseq and from TCGA datasets using the R package “clusterProfiler” (*p*-values were adjusted using the Benjamini–Hochberg procedure) [65]. GO, KEGG, and REACTOME enrichment analyses of differentially expressed genes derived from hTERT-HME1 WT and HOXA2^KO^ cells-mRNA sequencing were executed using clusterProfiler R package by Novogene. Terms and pathways with *p*-value less than 0.05 were considered significantly enriched within the list of differentially expressed genes.

### Micro/photograph analysis

Unless otherwise specified, images were processed using custom R scripts (https://www.r-project.org/) taking advantage of EBImage (https://www.bioconductor.org/) and MorphR software packages (https://github.com/kroemerlab/MorphR). Generally, grayscale images (either native or extracted from RGB components) were subjected to a sigmoidal transform and thereafter thresholded to obtain masks delimiting the regions of interest (ROI) from which morphometric features were computed.

### Statistical analysis for in vitro and in vivo experimentations

For in vitro data, significance was assessed by Student’s *t* or ANOVA (one-way or two-way). Each experiment was performed independently at least two times, and quantitative data were expressed as mean ± standard deviation (SD) or standard error of the mean (SEM). Statistical analyses and data graphing were performed using GraphPad® Prism software V. 10.2.3. Outlier values were identified using the ROUT test. A *p*-value ≤ 0.05 was considered statistically significant.

For longitudinal comparison of tumor growth curves in vivo, statistical analysis was performed by linear mixed-effects models using TumGrowth web tool (https://kroemerlab.shinyapps.io/TumGrowth/). The size of animal groups was estimated based on previous investigations [66]. Randomization was performed using the software RandoMice. One mouse (group = *HOXA2*^KO^) was identified as outlier using the ROUT test. Data graphing was performed using GraphPad® Prism.

## Supplementary information

Supplementary Figures

Supplementary Tables

Original data

## Data Availability

The datasets used in the current study for in silico analyses were published previously. RNA-seq and methylation data of BC patients from the TCGA program were downloaded from the website Genomic Data Commons (https://portal.gdc.cancer.gov). Previously published METABRIC gene expression data were reanalyzed. Survival curves were plotted using the online survival analysis tool “Kaplan-Meier Plotter” (https://kmplot.com), and the individual datasets used for the estimation of each survival curve are listed in Supplementary Table S14. All the other data that support the findings of this study are available from the corresponding authors upon request.
