# Supplementary material for: Epigenetic regulation of HOXA2 expression affects tumor progression and predicts breast cancer patient survival
Source: Cell Death Differ. 2025 Jan 20;32(4):730–44. doi: 10.1038/s41418-024-01430-2 (PMC11982354; doi:10.1038/s41418-024-01430-2)
Supplement: Supplementary file 1 — Supplementary Figures [file 41418_2024_1430_MOESM1_ESM.pdf]

SUPPLEMENTAL INFORMATION

Epigenetic regulation of *HOXA2* expression affects tumor progression and predicts breast cancer patient survival

Fatima Domenica Elisa De Palma*, Jonathan G. Pol*, Vincent Carbonnier, Sarah Adriana Scuderi, Deborah Mannino, Léa Montégut, Allan Sauvat, Maria Perez-Lanzon, Elisabet Uribe Carretero, Valentina Del Monaco, Mario Guarracino, Ilaria Granata, Raffaele Calogero, Donatella Montanaro, Gautier Stoll, Gerardo Botti, Massimiliano D'Aiuto, Alfonso Baldi, Valeria D'Argenio, Roderic Guigó, René Rezsohazy, Guido Kroemer, Maria Chiara Maiuri# and Francesco Salvatore#

* Equal contributors, co-first authors.

# Co-last authors.

Corresponding authors: Francesco Salvatore (FS), salvator@unina.it; Maria Chiara Maiuri (MCM), chiara.maiuri@crc.jussieu.fr; Fatima Domenica Elisa De Palma (FDEDP), depalma@ceinge.unina.it

Short title: *HOXA2* acts as a tumor suppressor gene in breast tissue

Supplementary Figures

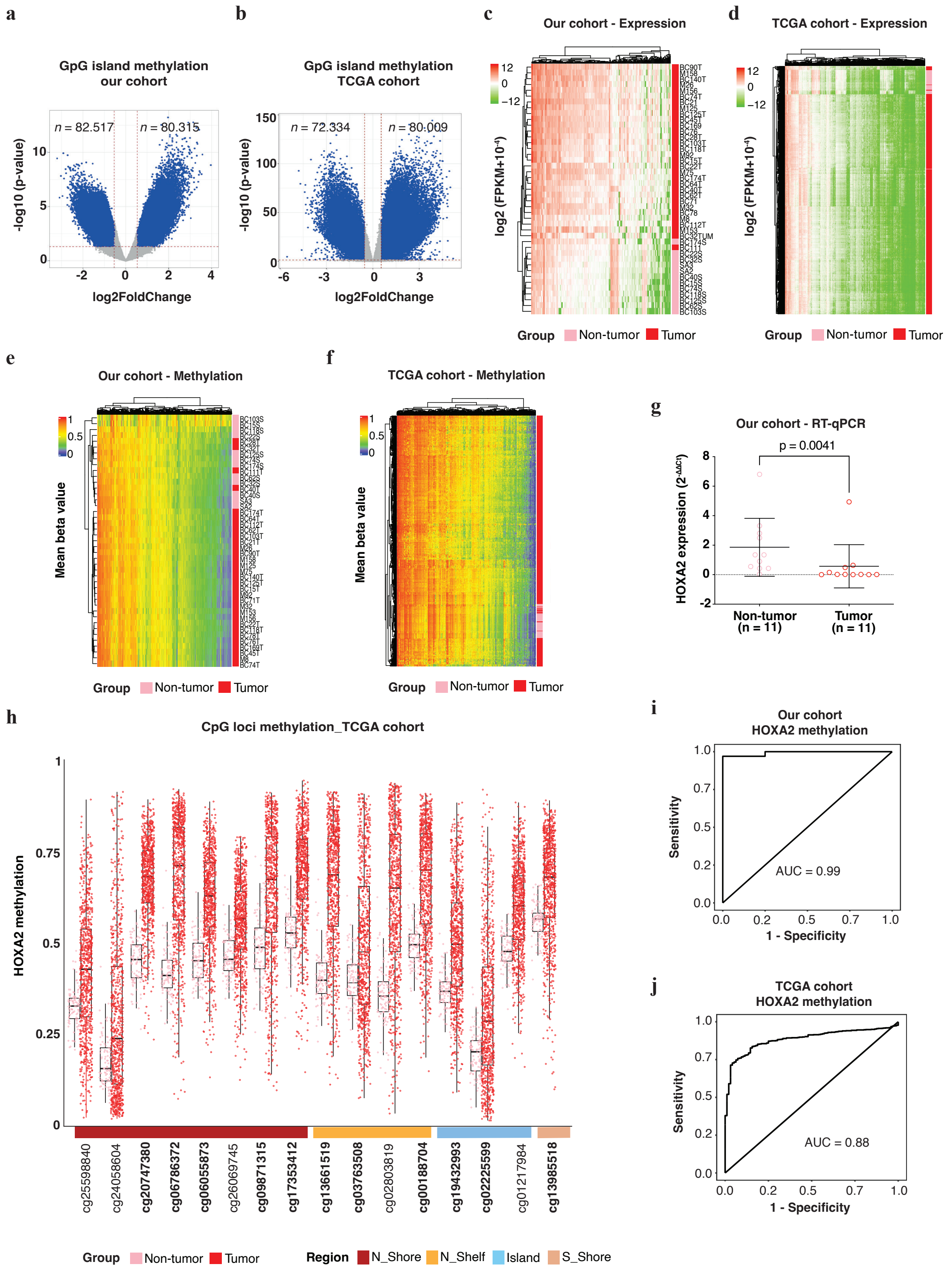

Supplementary Figure S1. Transcriptome and DNA methylome profiling revel reduced expression and promoter hypermethylation of *HOXA2* genes in breast cancer patients. (a, b) Volcano plots of differentially methylated CpG islands (reported according to their log2 fold change and log10 p-value) in non-tumor/normal versus tumor breast samples from our cohort (**a**) and from TCGA dataset (**b**). Of note, p-value ≤ 0.05 and $|\log_2 FC| \geq 0.5$ for significant differentially expressed CpG island were used as cut-off. Significant and unsignificant modulations are in blue and grey, respectively. **(c-f)** Heatmaps of genes differentially expressed with a $|\log_2 FC| \geq 1$ and a p-value $\leq 10^{-7}$ (**c**, **d**), or differentially methylated with a $|\log_2 FC| \geq 0.5$ and a p-value $\leq$

0.05 (**e**, **f**) from our cohort of BC samples (**c**, **e**) and from TCGA dataset (**d**, **f**). Each row represents a breast tissue sample whose type, i.e. non-tumor/normal or tumor, is color-coded. See **Supplementary Tables S1-S5** for details. (**g**) Dot plot illustrating the expression of *HOXA2* measured by RT-qPCR in a subset of breast tissues sequenced as in Panel 1c. Individual values expressed as $2^{-\Delta\Delta Ct}$ (*GAPDH,* the endogenous control) as well as means ± SD. Data were compared using Student's t test. (**h**) Boxplots showing differential level of methylation within different significant (p-value ≤ 0.05, |log2FC| ≥ 0.5) CpG loci of *HOXA2* in non-tumor/normal and breast tumor samples derived from TCGA dataset. *HOXA2* methylation levels were reported as beta values. CpG loci in common between our cohort (Figure 2n) and TCGA cohort (**h**) and showing the same significance (p-value ≤ 0.05, |log2FC| ≥ 0.5) are highlighted in bold. See **Supplementary Table S12** for details. (**i, j**) ROC curve analysis of the levels of *HOXA2* methylation in our cohort of breast patients (**i**) and in TCGA dataset (**j**). AUC, area under the curve; BC, breast cancer; CpG, Cytosine phosphate Guanine; FC, fold change; FPKM, fragments per kilobase of exon per million fragments mapped; ROC, receiver operating characteristic; TCGA, the cancer genome atlas.

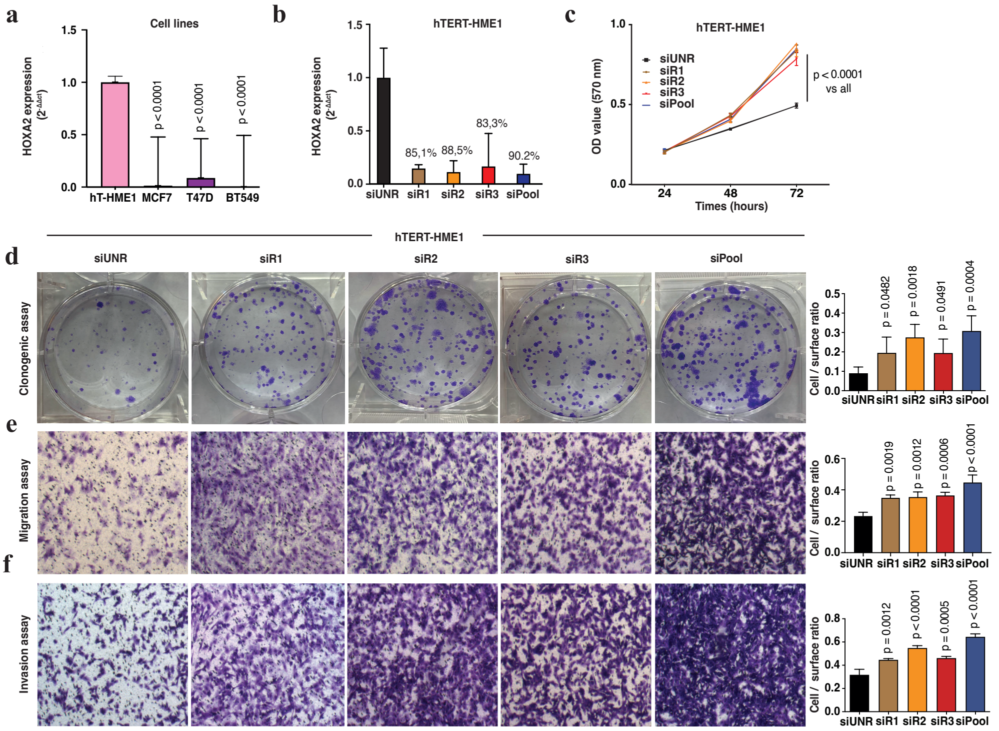

Supplementary Figure S2. Effect of *HOXA2* silencing in BC cell lines. (**a**) *HOXA2* expression in breast cell lines was measured by RT-qPCR and calculated according to the $2^{-\Delta\Delta Ct}$ (*GAPDH,* the endogenous control). (**b**) hTERT-HME1 cells were transiently transected with unrelated (siUNR) or 3 *HOXA2*-specific (siR1, siR2, siR3) or the pool of the 3 (siPool) siRNAs. The efficiency of *HOXA2* knockdown was evaluated by RT-qPCR. *GAPDH* was used as endogenous control. The % of knockdown was calculated as = (1-ΔΔCt) x100. (**c, d**) Cell proliferation in hTERT-HME1 cells following HOXA2 silencing was evaluated by (**c**) MTT assay and (**d**) clonogenic assay. (**e**, **f**) Cell migration (**e**) and cell invasion (**f**) were detected by transwell assay in absence of *HOXA2* in hTERT-HME1cells. (**d-f**) Left panels, representative images of colonies and transwell inserts stained with crystal violet. Right panels, corresponding quantification

of colony formation, and migration and invasion efficiency. (**a**-**f**) Data represent means ± SD from one representative experiment (a-c) and from 3 experiments (d-f). Significance by ANOVA (vs the corresponding control). hT-HME1, hTER-HME1 cell line; RT-qPCR, reverse-transcription quantitative real time PCR.

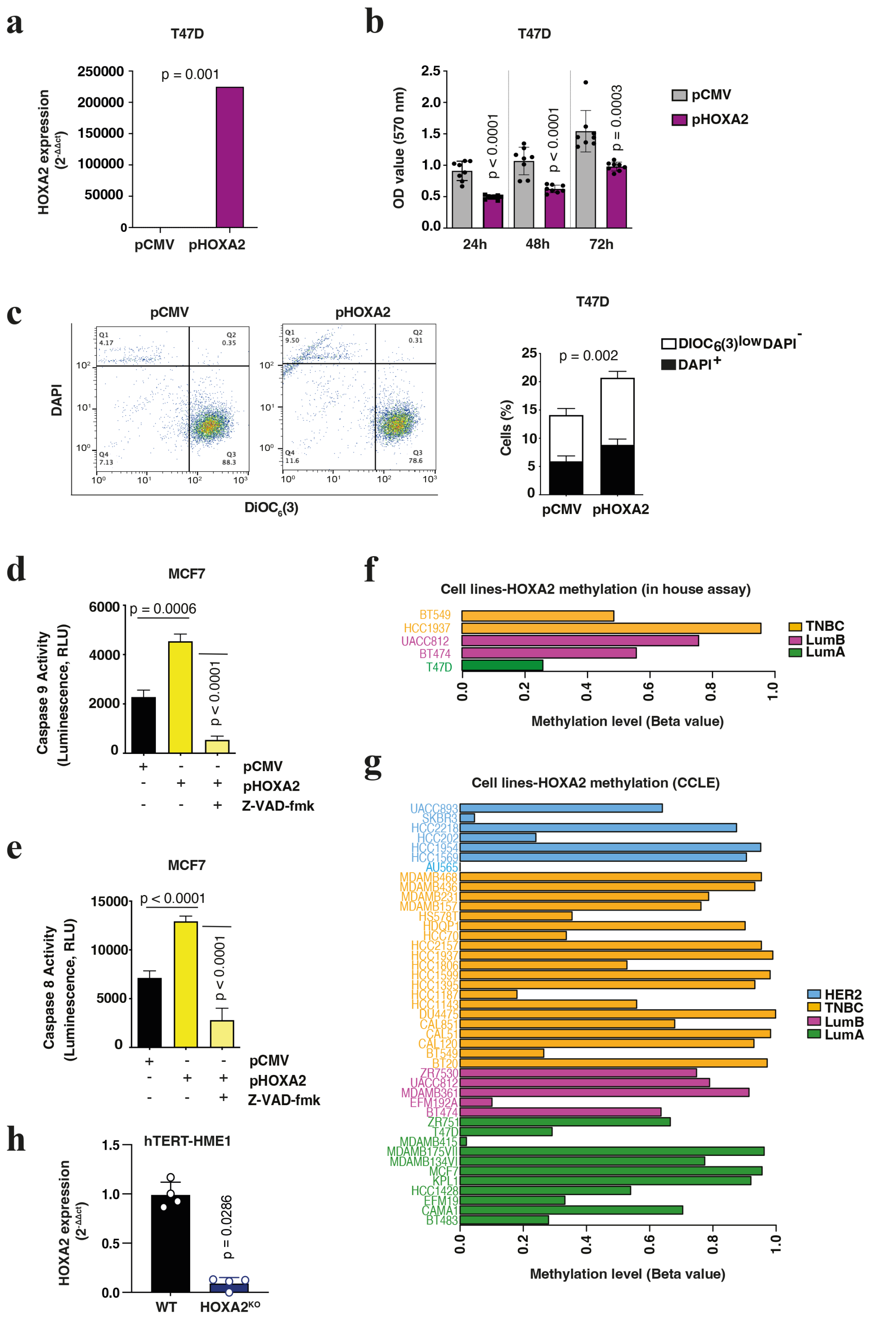

Supplementary Figure S3. ***HOXA2*** **deregulation in BC cells.** (**a**) *HOXA2* expression was measured by RT-qPCR in T47D cells transfected with *HOXA2* plasmid (pHOXA2) or the control vector (pCMV). *GAPDH* was used as endogenous control. (**b**) Cell proliferation was evaluated by MTT assay in T47D cells following *HOXA2* forced expression. Data represents means ± SD from one representative experiment. Statistical analyses were done using student's t test (vs corresponding control). (**c**) Effect of the overexpression of *HOXA2* on cell death by flow cytometry in T47D cells transfected with pCMV or pHOXA2 plasmids. Cells were subjected to a double staining with DAPI

and $DiOC_6(3)$ for the detection of dying ($DiOC_6(3)^{low}DAPI^{-}$) and dead ($DAPI^{+}$) cells. Representative flow cytometry plots (left panels) and statistical graph of the percentage of apoptotic and dead cells (right panel) are shown. Columns represent means ± SD. One representative experiment is shown. Statistical analyses were done using student's t test (vs corresponding control). (**d**, **e**) Caspase 9 and 8 activities after *HOXA2* overexpression in MCF7 cells. Quantification of caspase 9 (**d**) and caspase 8 (**e**) activity in MCF7 cells transfected with pCMV (control vector) or pHOXA2 (HOXA2 vector) in presence or absence of the pan-caspase Z-VAD-fmk (Z-VAD, 50 μM; 24h) was measured by luminescence. Data represents means ± SD of one representative experiment. Significance using Student's t test. (**f, g**) Bar plots show the methylation value of *HOXA2* measured in several human breast cell lines extracted from our in house Infinium 450k methylation array (**f**), and from the Cancer Cell Line Encyclopedia (CCLE) database (**g**). Values are reported as Beta value. (**h**) The efficiency of *HOXA2* knockout performed via CRISPR/Cas9-mediated system was measured in hTERT-HME1 *HOXA2* knockout ($HOXA2^{KO}$) or wildtype (WT) cells by RT-qPCR and calculated according to the $2^{-\Delta\Delta Ct}$ method (*GAPDH,* the endogenous control). CCLE, Cancer Cell Line Encyclopedia; HER2, human epidermal growth factor receptor 2; LumA, luminal A; LumB, luminal B; RLU, relative light units; RT-qPCR, reverse-transcription quantitative real time PCR; TNBC, triple-negative breast cancer.
