# Supplementary material for: Epigenetic regulation of HOXA2 expression affects tumor progression and predicts breast cancer patient survival
Source: Cell Death Differ. 2025 Jan 20;32(4):730–44. doi: 10.1038/s41418-024-01430-2 (PMC11982354; doi:10.1038/s41418-024-01430-2)

Original Western Blot
Figure 4, Panel q

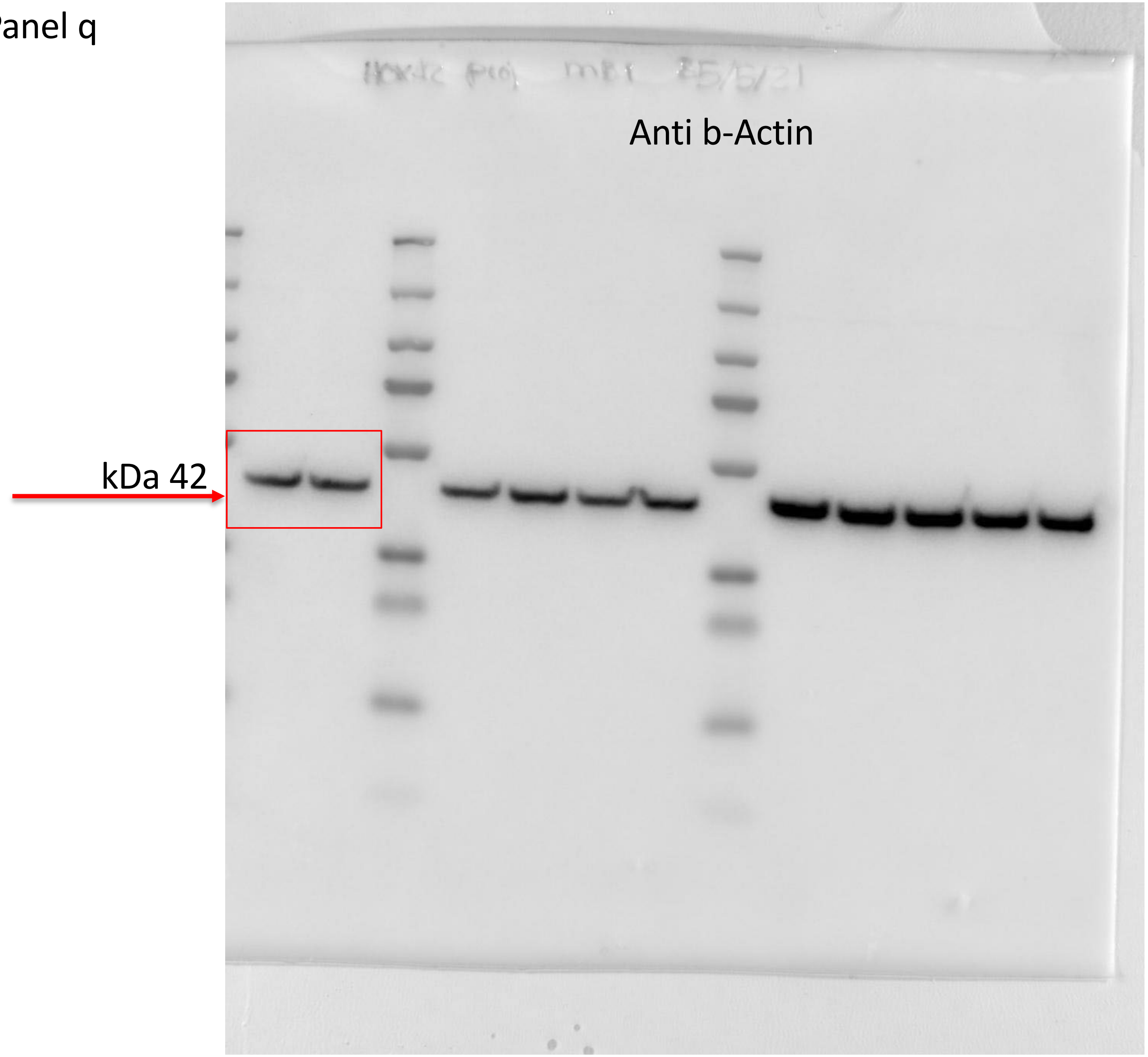

Original Western Blot
Figure 4, Panel q

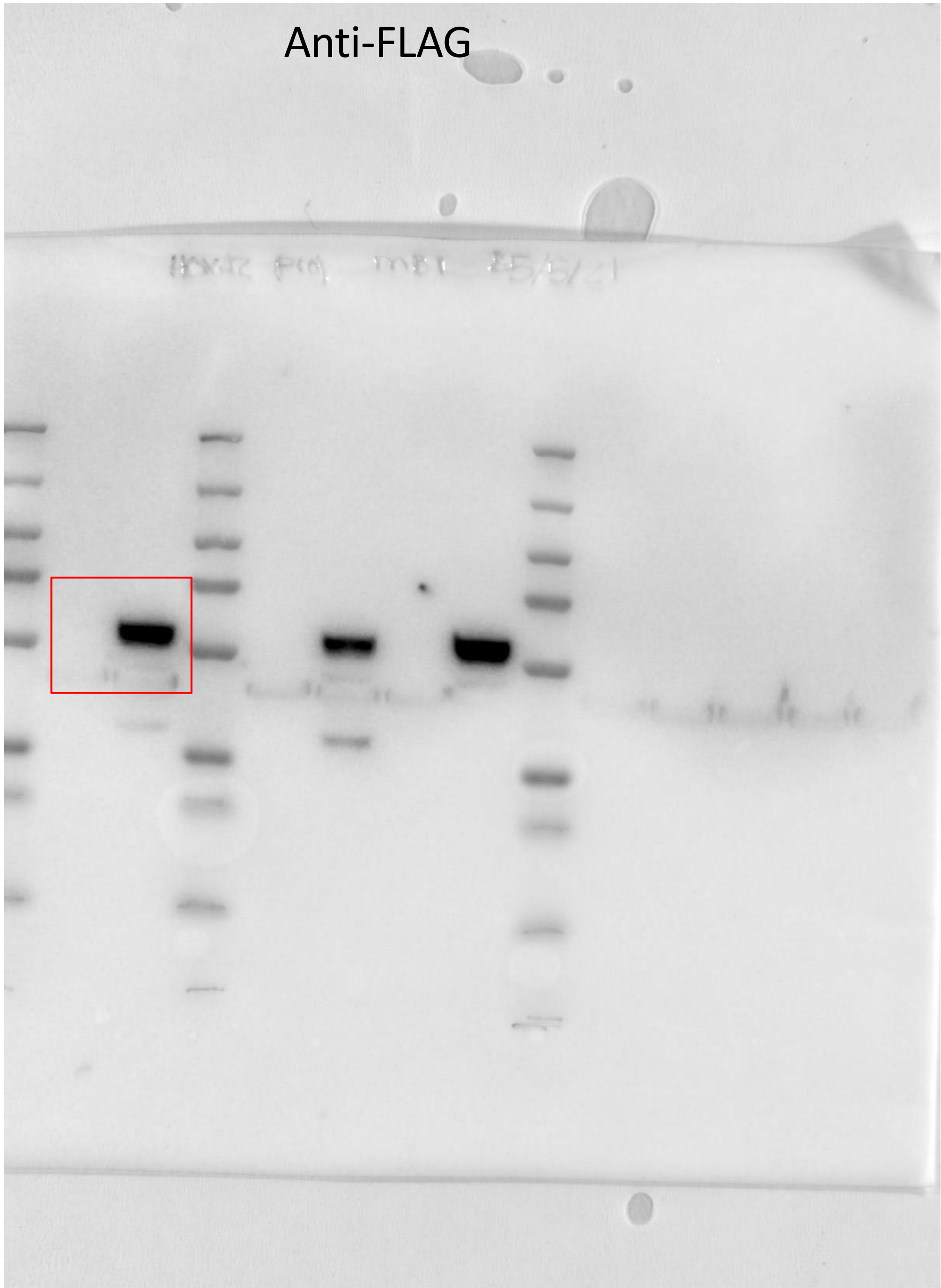

3X Flag-HOXA2 (42 kDa)

Original Western Blot
Figure 4, Panel q

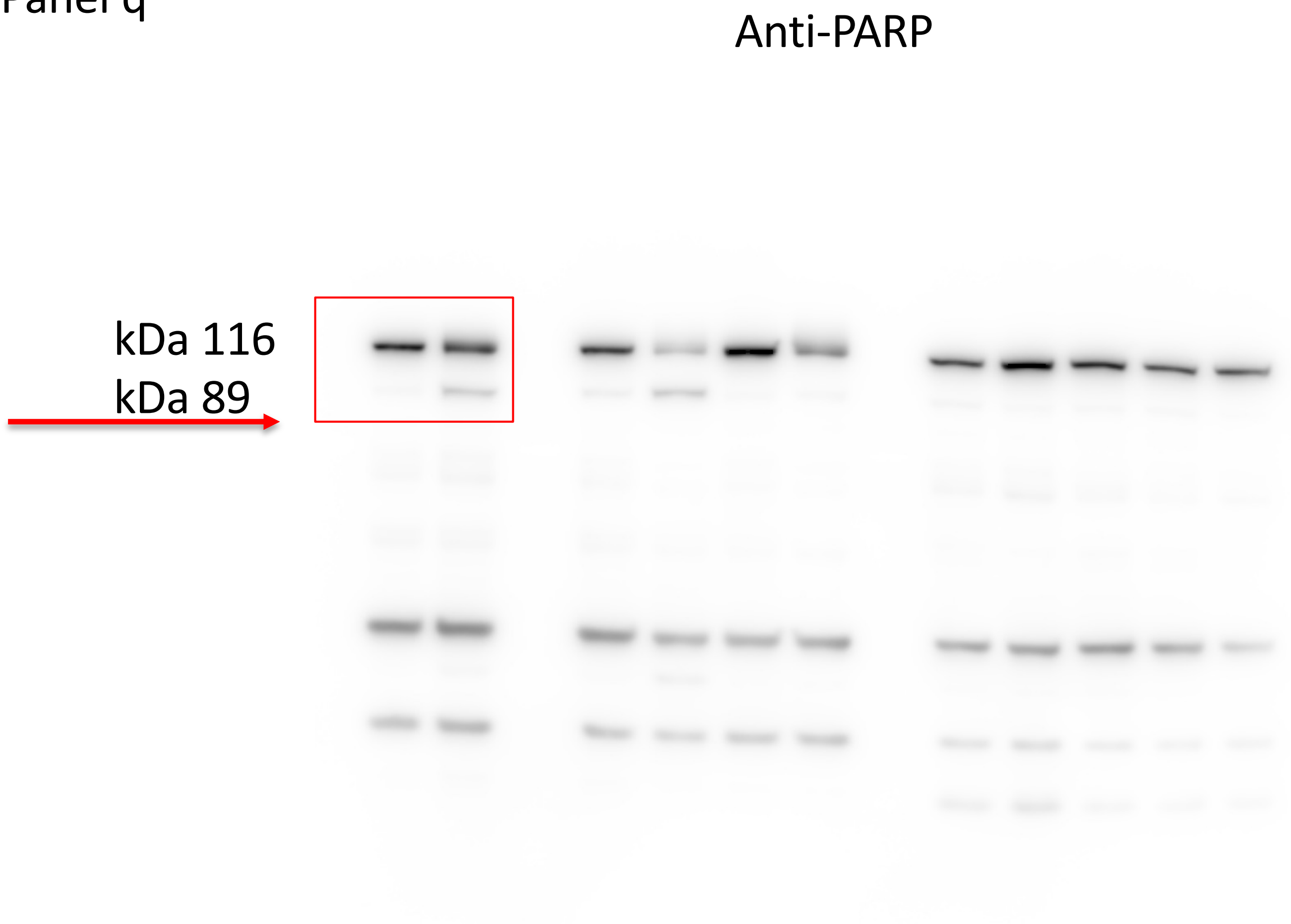

Original Western Blot
Figure 6, Panel d

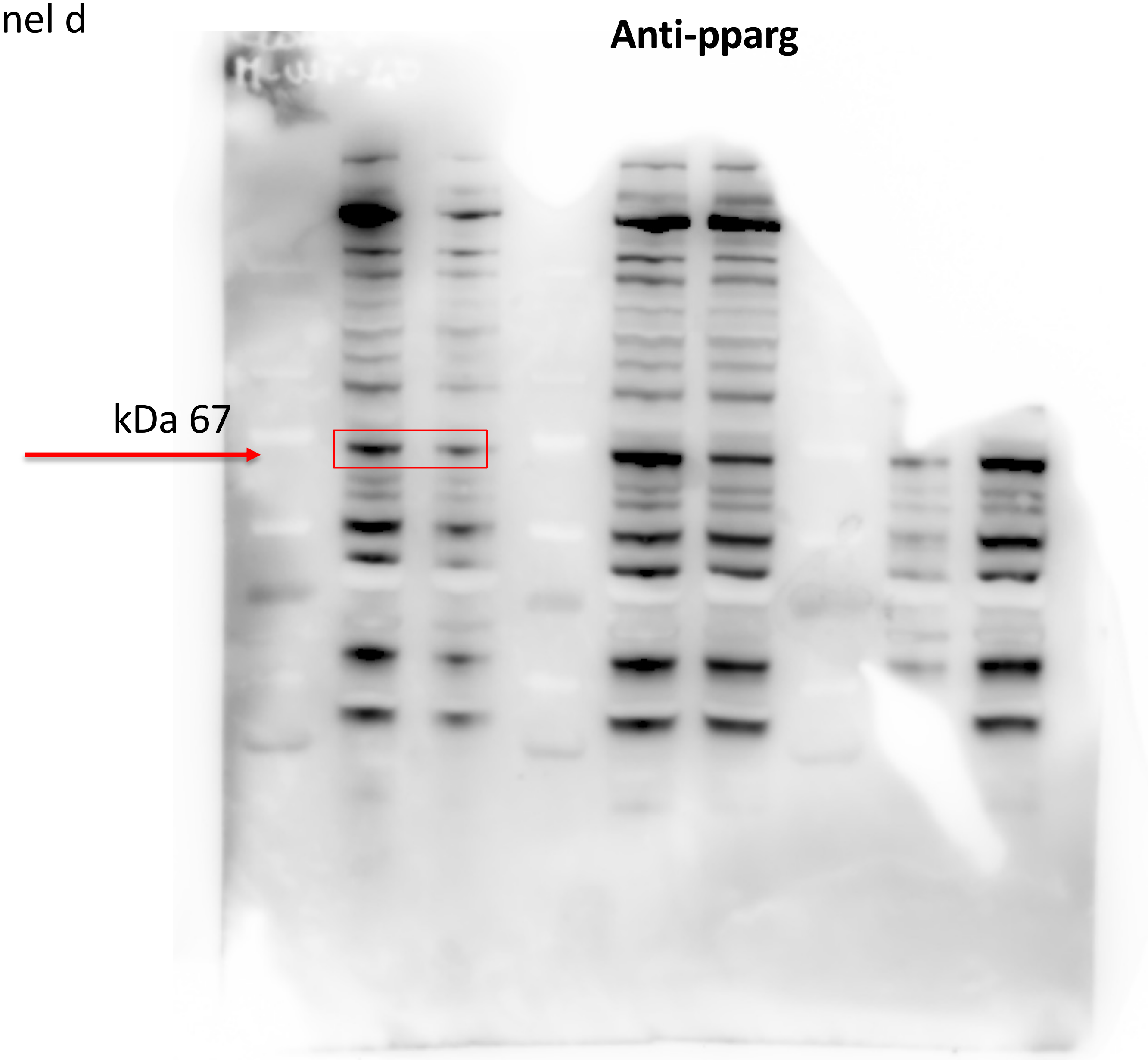

Original Western Blot
Figure 6, Panel d

Anti-CIDEC

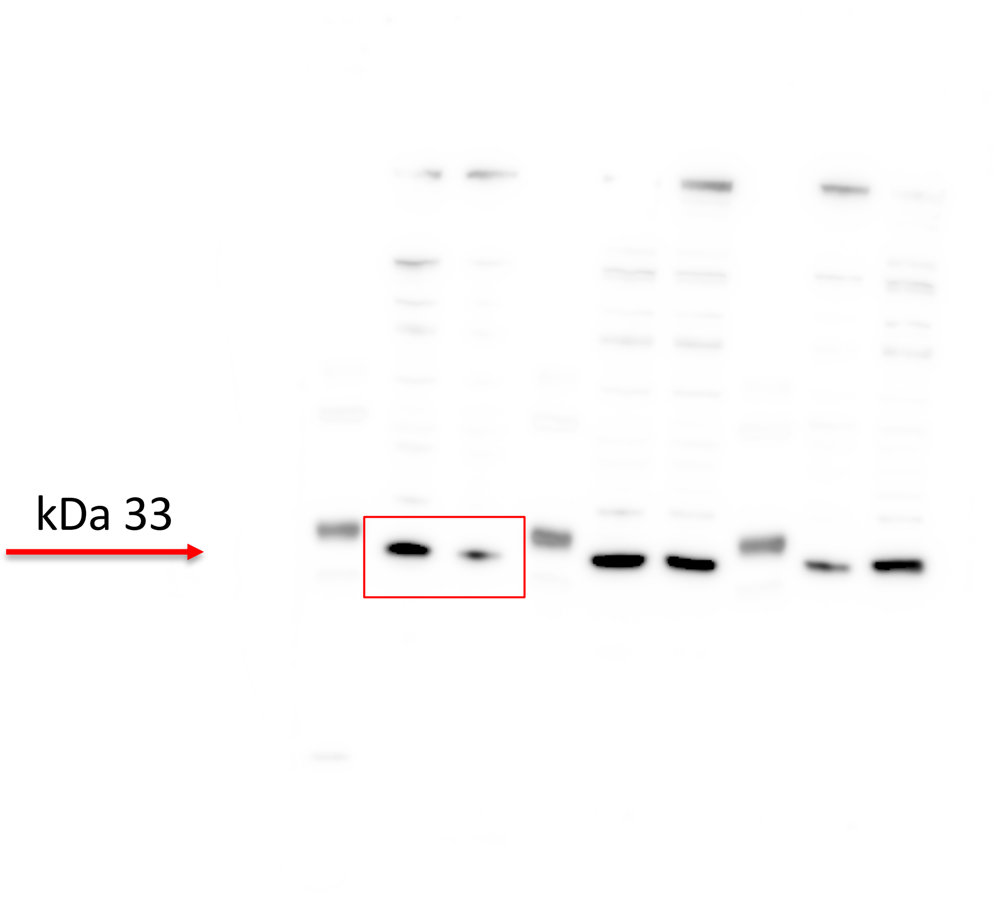

Original Western Blot
Figure 6, Panel d

Anti- B actin

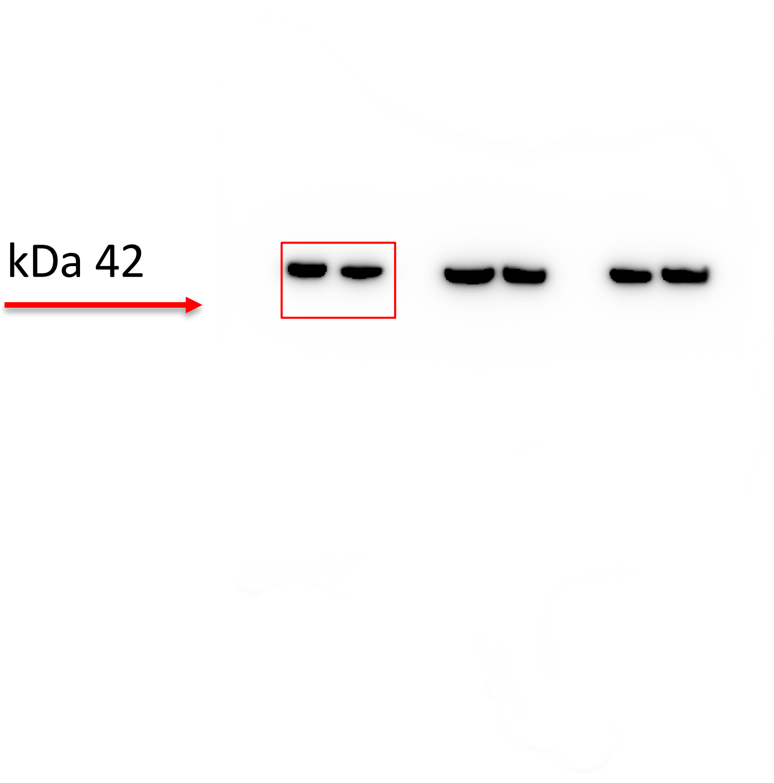

Original Western Blot
Figure 6e

Anti- Flag

3X Flag-HOXA2 (42kDa)

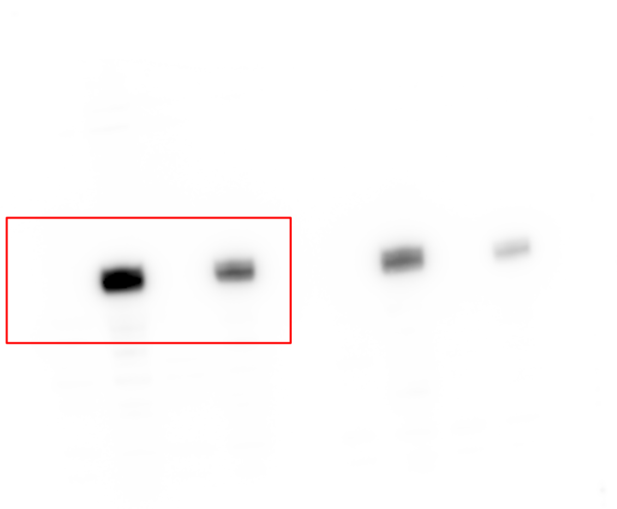

Original Western Blot
Figure 6e

Anti- PPARg

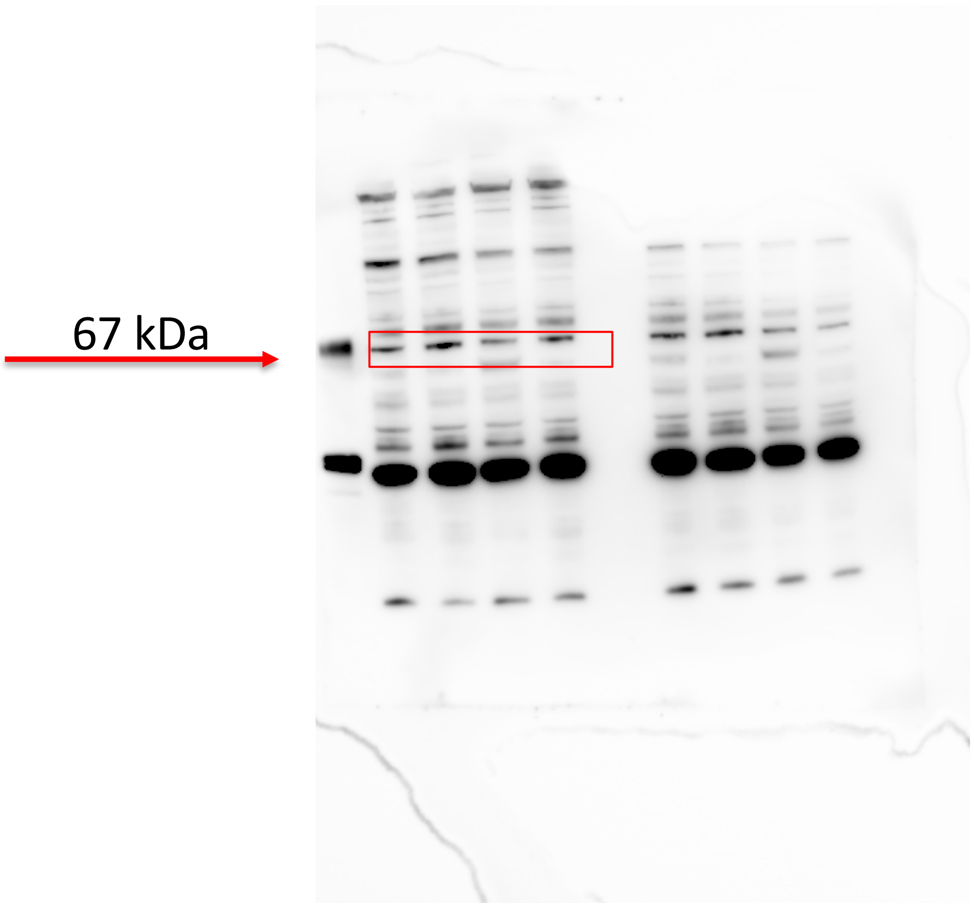

Original Western Blot
Figure 6e

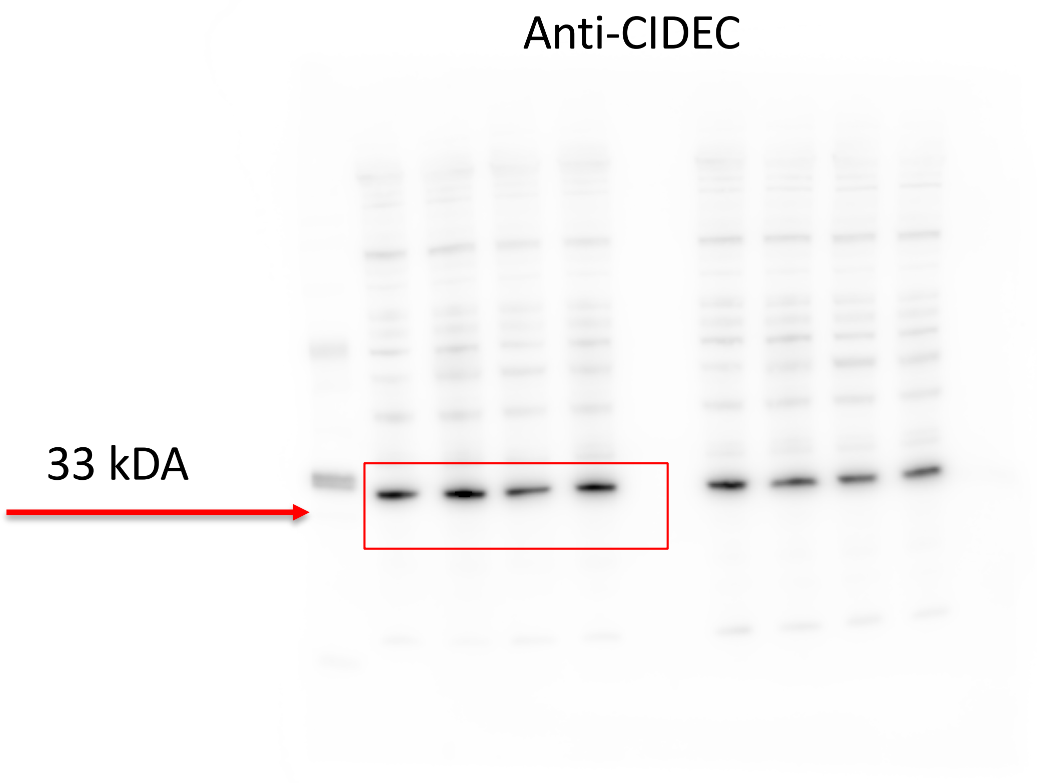

Original Western Blot
Figure 6e

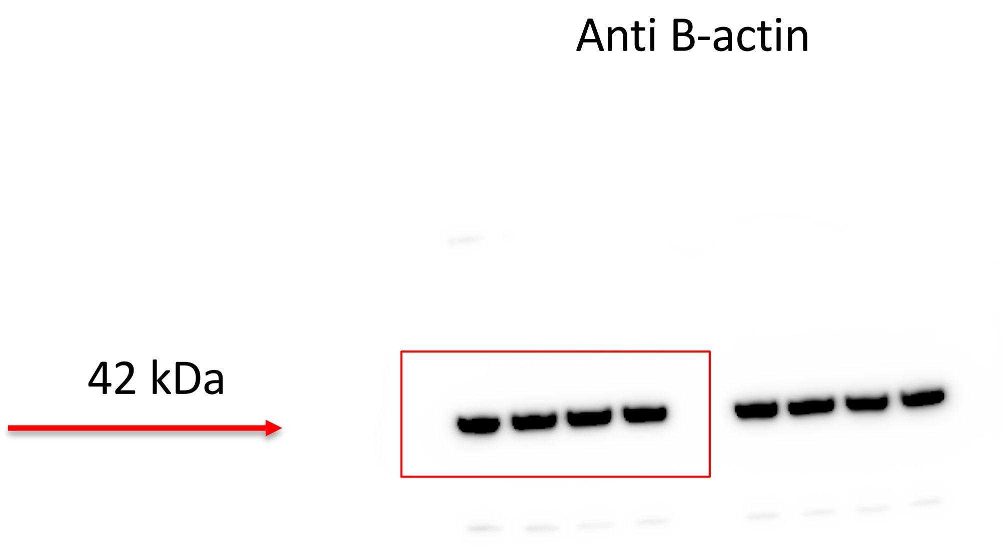

Original Picture of the representative dissected tumors from respective groups showed in Figure 5, Panel g

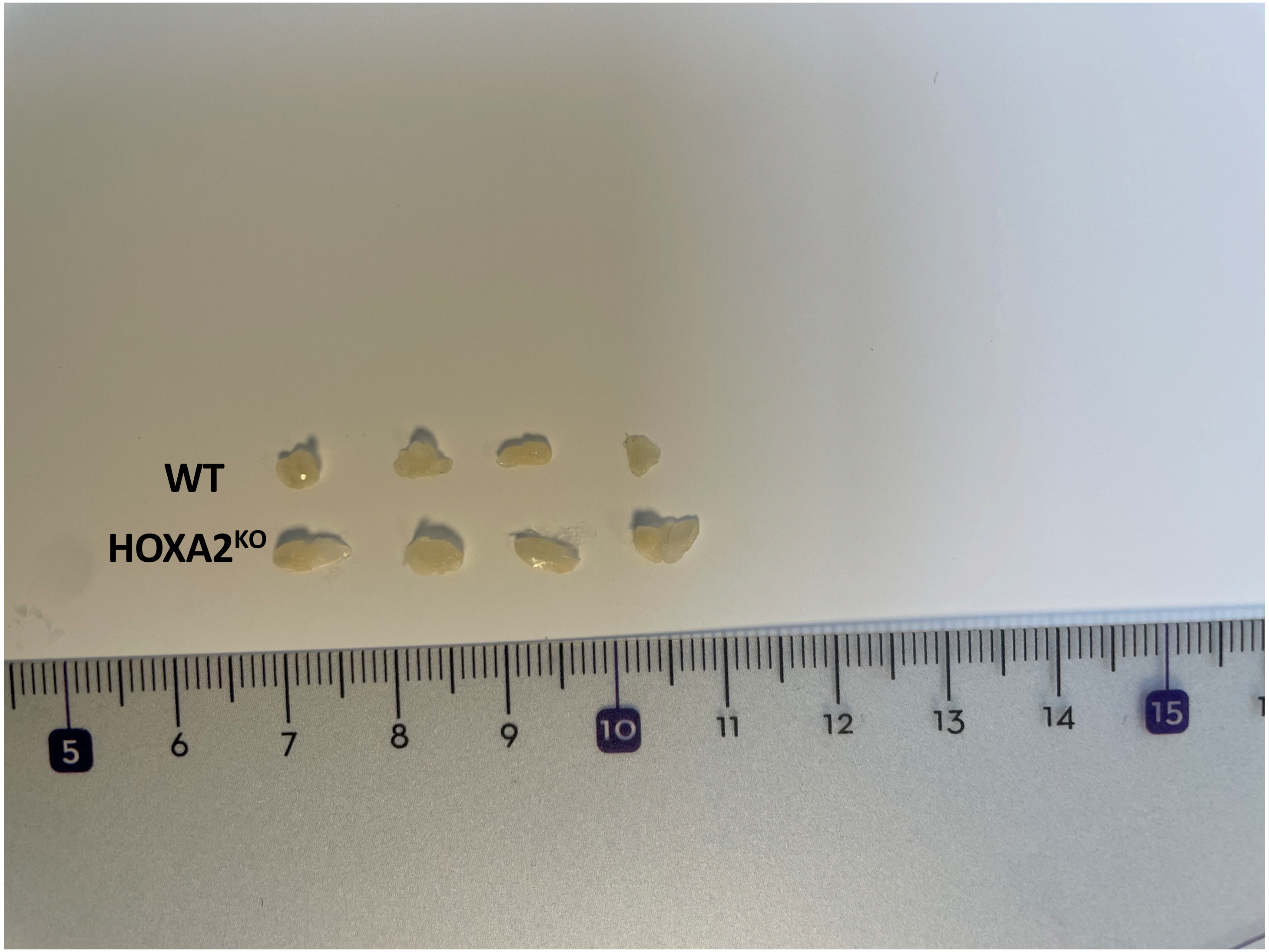

Supplement: Supplementary file 3 — Original data [file 41418_2024_1430_MOESM3_ESM.pdf]
